# Supplementary material for: Indicators of high-quality general practice to achieve Quality Equity and Systems Transformation in Primary Health Care (QUEST-PHC) in Australia: a Delphi consensus study
Source: PLoS One. 2025 Sep 5;20(9):e0327508. doi: 10.1371/journal.pone.0327508 (PMC12412998; doi:10.1371/journal.pone.0327508)
Supplement: S1 File — (PDF) [file pone.0327508.s001.pdf]

Supplementary file 1: 79 indicators and their associated 128 measures

Indicators	Related measures	
**ATTRIBUTE 1: ACCOUNTABLE TO OUR PATIENTS**		
**PERSON CENTRED CARE AND PATIENT-TEAM RELATIONSHIP**		1.
**S1: Availability of information for patients**	Written and electronic information in appropriate languages	2.
**P2: Patient input/feedback on health care delivery**	Evidence of formal process to consider patient input and incorporate into practice care delivery	3.
**O3: Patient perceptions of care**	Results of PREMs	4.
O4: Patient activation (blue sky)	*PAM® scores (blue sky)*	*5.*
O5: Strength of team- patient relationship (blue sky)	*Results from validated survey tool (blue sky)*	*6.*
**EVIDENCE-BASED COMPREHENSIVE CARE: PREVENTIVE HEALTH CARE**		
**P6: Risk factors recorded**	% active patients ≥15 years with a BMI recorded who have weight classification (obese, overweight, healthy, underweight) in previous 12 months	7.
	% active patients ≤ 15 years with height/length and weight recorded in previous 12 months	8.
	% active patients ≥15 years with a smoking status recorded/updated (current, ex-smoker, never smoked) in previous 24 months	9.
	% active patients ≥15 years with alcohol consumption status recorded in previous 24 months	10.
	% active patients aged 14-19 years with other substance use recorded	11.
	% active patients ≥18 years with BP recorded in previous 24 months	12.
P7: Childhood adverse experiences recorded (blue sky)	*% active patients aged 0-19 years screened for adverse childhood experiences in previous 12 months (blue sky)*	*13.*
**P8: Early detection of cancer**	% active patients aged 50-74 years with FOBT recorded in previous 24 months	14.
	% active female patients aged 25-74 years without hysterectomy with up-to-date cervical screening	15.
	% active female patients aged 50-74 years with no history of breast cancer screened with mammogram in previous 24 months (blue sky)	*16.*
**P9: Adult vaccination**	% active patients ≥65 years immunised against influenza in previous 15 months	17.
	% active patients with DM immunised against influenza in previous 15 months	18.
	% active patients with COPD ≥15 years immunised against influenza in previous 15 months	19.
	% active patients ≥70 years with one dose of pneumococcal immunisation recorded and for Aboriginal and Torres Strait Islander patients ≥50 years two doses at 5-year interval	20.
	% active patients >70-79 years with shingles vaccination	21.
**P10: Childhood vaccination**	% active patients ≥4 years who are fully immunised according to guidelines	22.
**P11: Aboriginal and Torres Strait Islander preventive health care**	% active patients identified as Aboriginal and/or Torres Strait Islander with Aboriginal Health Check in previous 15 months	23.
**O12: Patient perceptions of preventive health discussion**	PREMs to include patient report of discussion regarding the following health behaviours/risk factors: healthy eating	24.
	PREMs to include patient report of discussion regarding the following health behaviours/risk factors: exercise/physical activity	25.
	PREMs to include patient report of discussion regarding the following health behaviours/risk factors: risks of smoking/QUIT support,	26.
	PREMs to include patient report of discussion regarding the following health behaviours/risk factors: alcohol use	27.
	PREMs to include patient report of discussion regarding the following health behaviours/risk factors: unintentional injuries (home risk factors)	28.

	PREMs to include patient report of discussion regarding the following health behaviours/risk factors: unsafe sexual practices,	29.
	PREMs to include patient report of discussion regarding the following health behaviours/risk factors: unmanaged psychosocial stress	30.
**EVIDENCE-BASED COMPREHENSIVE CARE: CHRONIC CARE**		
**S13: Systems for management of chronic disease**	Use of patient chronic disease registers	31.
**P14: Systems for management of chronic disease**	Use of registers for patient follow up and recall	32.
**S15: Diabetes: known prevalence**	% of active patients with diabetes coded in patient records	33.
**P16 Diabetes: monitoring CV risk**	% active patients with DM and have their BP recorded in previous 6 months	34.
	% active patients with DM and have their BMI recorded	35.
	% active patients with T2DM and have their total Cholesterol, HDL, triglyceride and LDL levels recorded	36.
**P17: Diabetes: monitoring renal function**	% active patients with DM and have their eGFR (estimated glomerular filtration rate) recorded in previous 12 months	37.
	% active patients with DM and have their urine ACR recorded in previous 12 months	38.
**P18: Diabetes: managing risk**	% active patients >60 years with T2DM prescribed a statin	39.
**P19: Diabetes care: managing complications**	% active patients with DM and have their retinal screening performed in previous 24 months	40.
	% active patients with DM and have their diabetic foot assessment in previous 12 months	41.
**P20: Diabetes: monitoring blood sugar control**	% active patients with DM and have their HbA1c recorded in previous 12 months	42.
**O21: Diabetes: optimal management**	% active patients with T2DM with HbA1c ≤8%	43.
	% active patients with T2DM with BP <140/90 mmHg	44.
**O22: Diabetes: optimal risk management**	% active patients with T2DM with lipids to target in previous 12 months	45.
	% active patients with T2DM with microalbuminuria on ACE inhibitor or ARB	46.
	% active patients >16 years with DM and not smoking	47.
**S23: Respiratory disease: known prevalence**	% active patients with COPD coded in patient records	48.
	% active patients with asthma coded in patient records	49.
**P24: Respiratory disease: use of spirometry record**	% active patients with COPD and have spirometry	50.
	% active patients with asthma and have their spirometry recorded in previous 24 months	51.
**P25: Respiratory disease: monitoring risk factors**	% active patients with COPD and have their smoking status recorded	52.
	% active patients >15 years with asthma and have their smoking status recorded	53.
P26: Respiratory disease: planning care (blue sky)	*% active patients with asthma with an asthma management plan (blue sky)*	*54.*
P27: Respiratory disease: Control (blue sky)	*% active patients with COPD and have their COPD Assessment Test score recorded (blue sky)*	*55.*
	% active patients with asthma and have Asthma Control Questionnaire recorded (blue sky)	*56.*
**P28: Respiratory disease: appropriate use of medication**	% active patients with COPD on LAMA	57.
	% active patients ≥12 years with asthma on ICS containing preventer (blue sky)	*58.*
O29: Respiratory disease: COPD control (blue sky)	*% active patients with COPD and have been hospitalised in previous 6 months (blue sky)*	*59.*
**S30: Cardiovascular disease: known prevalence**	% active patients with CVD by category coded in patient records	60.
**P31: Cardiovascular disease: monitoring CVD risk**	% active patients aged 45 -74 years with the necessary risk factors assessed (smoking, diabetes, BP, Total Chol, HDL, age, gender) to enable CVD assessment in previous 24 months	61.
	% active patients aged 45-75 years with no known CVD and with absolute CVD risk calculated in previous 24 months	62.

	% active Aboriginal and/or Torres Strait Islander patients aged 35-75 years with no known CVD and with absolute CVD risk calculated in previous 24 months	63.
**P32: Cardiovascular disease: monitoring CVD**	% active patients ≥18 years with hypertension and have BP recorded in the previous 6 months	64.
**P33: Cardiovascular disease: management of CVD**	% active patients ≥18 years with CVD and have statin prescribed	65.
**O34: Cardiovascular disease: Optimal outcome**	% active patients with hypertension whose most recent BP is <140/90 mmHg	66.
**S35: Renal disease: known prevalence**	% active patients with renal disease coded in patient records	67.
**P36: Renal disease: screening for renal disease**	% active patients with DM screened for nephropathy (eGFR and ACR) in previous 12 months	68.
	% active patients coded in patient record as having hypertension screened for nephropathy (eGFR and ACR) in previous 12 months	69.
	% active Aboriginal and/or Torres Strait Islander patients >30 years screened for nephropathy (eGFR and ACR) in previous 24 months	70.
**P37: Renal disease: monitoring renal disease**	% active patients with renal disease and had their BP recorded in previous 12 months	71.
	% active patients with renal disease and had their eGFR recorded in previous 12 months	72.
	% active patients with renal disease and had their urine ACR recorded in previous 12 months	73.
	% active patients with renal disease and had their chronic kidney disease stage recorded	74.
**O38: Renal disease: dialysis**	% active patients with renal disease on dialysis	75.
**S39: Mental health: known prevalence of mental health conditions**	% active patients with mental health conditions within each mental health category	76.
**S40: Mental health: known prevalence of co-morbidity**	% active patients with mental health and also diagnosed with each of following: diabetes, CVD, respiratory and renal disease	77.
**P41: Mental health: treatment planning**	% active patients with mental health with a GP Mental Health Treatment Plan (such as MBS item number 2715) in previous 12 months	78.
**P42: Mental health: management of patients with a mental health diagnosis documented**	% active patients ≥15 years with a BMI recorded who have weight classification (obese, overweight, healthy, underweight) in previous 12 months	79.
	% active patients ≥15 years with a smoking status recorded/ updated (current, ex-smoker, never smoked) in previous 24 months	80.
	% active patients ≥15 years with alcohol consumption status recorded in previous 24 months	81.
	% active patients with follow-up GP visit within 7-30 days of hospital discharge related to psychiatric condition (blue sky)	*82.*
S43: Advance care planning (blue sky)	*% active patients ≥75 years with discussions about advance care planning recorded on file (blue sky)*	*83.*
P44: Advance care planning (blue sky)	*% active patients ≥75 years with Advance Care Plan uploaded to My Health Record (blue sky)*	*84.*
**ACUTE CARE: PRESCRIBING SAFETY**		
**S45: Safe prescribing of opioids and benzodiazepines**	Practice has a policy on the safe prescription of opioids and BZDs	85.
**S46: Safe prescribing of opioids and benzodiazepines**	Practice has a policy on discussing safe prescription of opioids and BZDs with all new prescribers	86.
**O47: Safe prescribing of opioids and benzodiazepines**	% acute patients prescribed opioids who had discussion of risk of opioid use with prescriber	87.

ATTRIBUTE 2: PROFESSIONALLY ACCOUNTABLE		
**MULTIDISCIPLINARY TEAM-BASED CONTINUING CARE THAT IS COORDINATED AND INTEGRATED WITH OTHER SERVICES AND THE MEDICAL NEIGHBOURHOOD**		
**S48: Practice goal/mission**	Defined practice mission/goal	88.
	Mission/goal accessible to staff	89.
	Mission/goal accessible to patients	90.
**S49: Practice profile**	Total number of staff in each professional category including FTE	91.
**S50: Data sharing with local hospitals**	Able to receive electronic discharge summary	92.
	Able to receive data in real time e.g. shared EHR or real time electronic shared care plan (blue sky)	*93.*
**S51: Data sharing with other health care providers**	*Practice has a system for notifying GPs of specialist and allied health care correspondence ((blue sky)*	*94.*
**S52: Use of My Health Record**	% of active patients with Shared Health summaries uploaded to My Health Record	95.
**P53: Team-based care**	Regular clinical review meetings involving all team members	96.
	Assigned care teams to coordinate care for individual patients (blue sky)	*97.*
	Reports from each team member in patient file	98.
**P54: Care planning**	% active patients with chronic disease who had a GP management plan in previous 12 months	99.
	% active patients with chronic disease who had a medication management review (HMR) in previous 12 months	100.
**O55: GP and staff satisfaction**	Survey measuring GP and staff satisfaction with: enjoyment of work	101.
	Survey measuring GP and staff satisfaction with: impact on local community health	102.
	Survey measuring GP and staff satisfaction with: safety in work	103.
	Survey measuring GP and staff satisfaction with: income from work	104.
	Survey measuring GP and staff satisfaction with: time with patients	105.
	Survey measuring GP and staff satisfaction with: work/life balance	106.
O56: Patient experience of continuity of care (blue sky)	*PREM questions on time taken for notification of abnormal test results (blue sky)*	*107.*
**O57: Care plan engages patient**	PREM questions on experience with care planning	108.
	PAM® scores (blue sky)	109.
O58: Follow-up following hospital attendance (blue sky)	*% of active patients reviewed following* ED presentation within 7 days *(blue sky)*	*110.*
	% of active patients reviewed following admission within 3 days (blue sky)	*111.*
**CLINICAL GOVERNANCE**		
**S59: Clinical governance systems in place**	Practice currently accredited according to RACGP or ACRRM standards	112.
**STAFF TRAINING**		
**P60: Regular staff education undertaken**	Number of meetings/attendances recorded	113.
**P61: Assessment of learning needs**	Evidence of process for assessment of learning needs	114.
**DATA-ENABLED PRACTICE QUALITY IMPROVEMENT**		
	% active patients with date of birth recorded	115.

**S62: Data quality and completeness of demographic and key health data**	% active patients with gender recorded	116.
	% active patients with allergy or 'nil known allergy' coded in patient records	117.
**P63: Improving the quality of our practice**	Evidence of work on data cleansing	118.
	Data reports and date of most recent report	119.
	Evidence of formal review of the collected data	120.
O64: Consumer satisfaction with quality of care (blue sky)	*Analysis of validated survey responses (PREMs) (blue sky)*	*121.*
**EDUCATION, TRAINING AND RESEARCH TO SUPPORT QUALITY AND SUSTAINABILITY**		
**S65: Registered for postgraduate GP training**	Accredited as training practice with local RTO	122.
**P66: Engagement with student training**	Number of medical, nursing and allied health students undertaking placements in previous 12 months	123.
P67: Research activity (blue sky)	*Evidence of engagement with research or PDSA activities (blue sky)*	*124.*
**ATTRIBUTE 3: ACCOUNTABLE TO THE COMMUNITY**		
**S68: Urgent access to care**	Provides same day appointments	125.
**S69: Access to non-face-to-face care e.g. telephone, email**	Process documented and advertised to patients for phone/email access	126.
**S70: Patient demographics recorded**	% active patients with cultural and linguistic status recorded	127.
	% active patients who identify as Aboriginal and/or Torres Strait Islander	128.
	% active patients with Aboriginal and/or Torres Strait Islander status coded in patient records	129.
	% active patients ≥16 years with Australian Government health care card	130.
**S71: Meets the needs of Aboriginal and/or Torres Strait Islander patients**	Practice registered for PIP Indigenous Health Incentive	131.
**S72: Health related social needs assessed**	*% active patients with screening for health-related social needs recorded (blue sky)*	*132.*
**S73: Community engagement**	Practice has community/patient advisory structures	133.
**P74: Provides health care to vulnerable communities**	Bulk billing for Australian Government health care card holders	134.
**P75: Meets the needs of CALD communities**	Provides bilingual services as required	135.
**O76: Access to regular primary care provider (as measured in response to PREMs)**	*% active patients reporting they have a specific GP/ practice nurse/ care team (blue sky)*	*136.*
	% active patients reporting difficulties obtaining care in previous 12 months	137.
	% active patients reporting same day response to phone call to GP/ nurse	138.
**O77: Access for low SES**	*Compare % active patients who are* Australian Government *health care card holders with % holding* Australian Government *health care cards in practice LGA (blue sky)*	*139.*
**ATTRIBUTE 4: ACCOUNTABLE TO SOCIETY**		
O78: Avoidable hospital care (blue sky)	*Use of linked data to measure potentially preventable hospital admissions (blue sky)*	*140.*

O79: Duplication of care (blue sky)	*Use of linked data to avoid duplication of pathology and radiology services (blue sky)*	*141.*

Footnote: **S** = structural indicators measuring organisation factors that define the health system including material resources (e.g. facilities, equipment, money), human resources (e.g. number and qualifications of staff) and organisation structure (e.g. staff organisation, methods of reimbursement); **P** = process indicators measuring what is actually done in giving and receiving care and can also be thought of as activities; **O** = outcome indicators measuring the effect of care on populations and patients.
