# Supplementary material for: Indicators of high-quality general practice to achieve Quality Equity and Systems Transformation in Primary Health Care (QUEST-PHC) in Australia: a Delphi consensus study
Source: PLoS One. 2025 Sep 5;20(9):e0327508. doi: 10.1371/journal.pone.0327508 (PMC12412998; doi:10.1371/journal.pone.0327508)
Supplement: S2 File — (PDF) [file pone.0327508.s002.pdf]

Supplementary file 2: Practice staff and PHN staff comparison on the feasibility of the indicators and

Indicators and measures

**ROUND 1**
**ATTRIBUTE 1**
Indicator S1: Availability of information for patients - Feasibility - Measure S1a: Written and electronic information in
Indicator P2: Patient input/feedback on health care delivery - Feasibility - Measure P2a: Evidence of formal process to consider patient input and incorporate into practice care delivery
Indicator O3: Patient perceptions of care - Feasibility - Measure O3a: Results of Patient Reported Experience
Indicator O4: Patient activation - Feasibility - Measure O4a: Patient Activation Measure® scores
Indicator O5: Strength of team-patient relationship - Feasibility - Measure O5a: Results from validated survey tool
Indicator P6: Risk factors recorded - Feasibility - Measure P6a: % active patients ≥15 years with a BMI recorded who have weight classification (obese, overweight, healthy, underweight) in previous 12 months
Indicator P6: Risk factors recorded - Feasibility - Measure P6b: % active patients ≤15 years with height/length and weight recorded in previous 12 months
Indicator P6: Risk factors recorded - Feasibility - Measure P6c: % active patients ≥15 years with a smoking status recorded/updated (current, ex-smoker, never smoked) in previous 24 months
Indicator P6: Risk factors recorded - Feasibility - Measure P6d: % active patients ≥15 years with alcohol consumption status recorded in previous 24 months
substance use recorded
Indicator P6: Risk factors recorded - Feasibility - Measure P6f: % active patients ≥18 years with BP recorded in
Indicator P7: Childhood adverse experiences recorded - Feasibility - Measure P7a: % active patients aged 0-19 years screened for adverse childhood experiences in previous 12 months
Indicator P8: Early detection of cancer - Feasibility - Measure P8a: % active patients aged 50-74 years with FOBT recorded in previous 24 months
Indicator P8: Early detection of cancer - Feasibility - Measure P8b: % active female patients aged 25-74 years without hysterectomy with up-to-date cervical screening
Indicator P8: Early detection of cancer - Feasibility - Measure P8c: % active female patients aged 50-74 years with no history of breast cancer screened with mammogram in previous 24 months
Indicator P9: Adult vaccination - Feasibility - Measure P9a: % active patients ≥65 years immunised against influenza in
Indicator P9: Adult vaccination - Feasibility - Measure P9b: % active patients with DM immunised against influenza in
Indicator P9: Adult vaccination - Feasibility - Measure P9c: % active patients with COPD ≥15 years immunised against influenza in previous 15 months
pneumococcal immunisation recorded and for Aboriginal and Torres Strait Islander patients ≥50 years two doses at 5-year interval
Indicator P9: Adult vaccination - Feasibility - Measure P9e: % active patients >70-79 years with shingles vaccination
Indicator P10: Childhood vaccination - Feasibility - Measure P10a: % active patients ≥4 years who are fully immunised
Indicator P11: Aboriginal and Torres Strait Islander preventative health care - Feasibility - Measure P11a: % active patients identified as Aboriginal or Torres Strait Islander with Aboriginal health check in previous 15 months
Indicator O12: Patient perceptions of preventative health discussion - Patient Reported Experienc... - Feasibility - Measure O12a: Healthy eating
Indicator O12: Patient perceptions of preventative health discussion - Patient Reported Experienc... - Feasibility - Measure O12b: Exercise/physical activity
Indicator O12: Patient perceptions of preventative health discussion - Patient Reported Experienc... - Feasibility - Measure O12c: Risks of smoking/quit smoking
Indicator O12: Patient perceptions of preventative health discussion - Patient Reported Experienc... - Feasibility -
Indicator O12: Patient perceptions of preventative health discussion - Patient Reported Experienc... - Feasibility - Measure O12e: Unintentional injuries (home risk factors)

Indicator O12: Patient perceptions of preventative health discussion - Patient Reported Experienc... - Feasibility - Measure O12f: Unsafe sexual practices
Indicator O12: Patient perceptions of preventative health discussion - Patient Reported Experienc... - Feasibility - Measure O12g: Unmanaged psychosocial stress
Indicator S13: Systems for management of chronic disease - Feasibility - Measure S13a: Use of patient chronic
Indicator P14: Systems for management of chronic disease - Feasibility - Measure P14a: Use of registers for patient
Indicator S15: Diabetes - known prevalence - Feasibility - Measure S15a: % of active patients with diabetes coded in
Indicator P16: Diabetes - monitoring CV risk - Feasibility - Measure P16a: % active patients with DM and have their BP recorded in previous 6 months
Indicator P16: Diabetes - monitoring CV risk - Feasibility - Measure P16b: % active patients with DM and have their
Indicator P16: Diabetes - monitoring CV risk - Feasibility - Measure P16c: % active patients with T2DM and have their total cholesterol, HDL, triglyceride and LDL levels recorded
Indicator P17: Diabetes - monitoring renal function - Feasibility - Measure P17a: % active patients with DM and have their eGFR recorded in previous 12 months
Indicator P17: Diabetes - monitoring renal function - Feasibility - Measure P17b: % active patients with DM and have their urine ACR recorded in previous 12 months
Indicator P18: Diabetes - managing risk - Feasibility - Measure P18a: % active patients >60 years with T2DM
Indicator P19: Diabetes - managing complications - Feasibility - Measure P19a: % active patients with DM and have their retinal screening performed in previous 24 months
Indicator P19: Diabetes - managing complications - Feasibility - Measure P19b: % active patients with DM and have their diabetic foot assessment in previous 12 months
Indicator P20: Diabetes - monitoring blood sugar control - Feasibility - Measure P20a: % active patients with DM and have their HbA1c recorded in previous 12 months
Indicator O21: Diabetes - optimal management - Feasibility - Measure O21a: % active patients with T2DM with
Indicator O21: Diabetes - optimal management - Feasibility - Measure O21b: % active patients with T2DM with BP
Indicator O22: Diabetes - optimal risk management - Feasibility - Measure O22a: % active patients with T2DM with lipids to target in previous 12 months
Indicator O22: Diabetes - optimal risk management - Feasibility - Measure O22b: % active patients with T2DM with microalbuminuria on ACE inhibitor or ARB
Indicator O22: Diabetes - optimal risk management - Feasibility - Measure O22c: % active patients >16 years with DM
Indicator S23: Respiratory disease - known prevalence - Feasibility - Measure S23a: % active patients with COPD
Indicator S23: Respiratory disease - known prevalence - Feasibility - Measure S23b: % active patients with asthma
Indicator P24: Respiratory disease - use of spirometry record - Feasibility - Measure P24a: % active patients with
Indicator P24: Respiratory disease - use of spirometry record - Feasibility - Measure P24b: % active patients with asthma and have their spirometry recorded in previous 24 months
Indicator P25: Respiratory disease - monitoring risk factors - Feasibility - Measure P25a: % active patients with COPD and have their smoking status recorded
Indicator P25: Respiratory disease - monitoring risk factors - Feasibility - Measure P25b: % active patients >15 years with asthma and have their smoking status recorded
Indicator P26: Respiratory disease - planning care - Feasibility - Measure P26a: % active patients with asthma with an asthma management plan
Indicator P27: Respiratory disease - control - Feasibility - Measure P27a: % active patients with COPD and have their COPD Assessment Test score
Indicator P27: Respiratory disease - control - Feasibility - Measure P27b: % active patients with asthma and have Asthma Control Questionnaire recorded
Indicator P28: Respiratory disease - appropriate use of medication - Feasibility - Measure P28a: % active patients
Indicator P28: Respiratory disease - appropriate use of medication - Feasibility - Measure P28b: % active patients ≥12 years with asthma on ICS containing preventer
Indicator O29: Respiratory disease - COPD control - Feasibility - Measure O29a: % active patients with COPD and have been hospitalised in previous 6 months

Indicator S30: Cardiovascular disease - known prevalence - Feasibility - Measure S30a: % active patients with CVD by category coded in patient records
years with the necessary risk factors assessed (smoking, diabetes, BP, Total Chol, HDL, age, gender) to enable CVD assessment in previous 24 months
Indicator P31: Cardiovascular disease - monitoring CVD risk - Feasibility - Measure P31b: % active patients aged 45-75 years with no known CVD and with absolute CVD risk calculated in previous 24 months
Strait Islander patients aged 35-75 years with no known CVD and with absolute CVD risk calculated in previous 24 months
Indicator P32: Cardiovascular disease - monitoring CVD - Feasibility - Measure P32a: % active patients ≥18 years with hypertension and have BP recorded in the previous 6 months
Indicator P33: Cardiovascular disease - management of CVD - Feasibility - Measure P33a: % active patients ≥18 years with CVD and have statin prescribed
Indicator O34: Cardiovascular disease - optimal outcome - Feasibility - Measure O34a: % active patients with hypertension whose most recent BP is <140/90 mmHg
Indicator S35: Renal disease - known prevalence - Feasibility - Measure S35a: % active patients with renal disease
Indicator P36: Renal disease - screening for renal disease - Feasibility - Measure P36a: % active patients with DM screened for nephropathy (eGFR and ACR) in previous 12 months
Indicator P36: Renal disease - screening for renal disease - Feasibility - Measure P36b: % active patients coded in patient record as having hypertension screened for nephropathy (eGFR and ACR) in previous 12 months
Indicator P36: Renal disease - screening for renal disease - Feasibility - Measure P36c: % active Aboriginal and/or Torres Strait Islander patients >30 years screened for nephropathy (eGFR and ACR) in previous 24 months
Indicator P37: Renal disease - monitoring renal disease - Feasibility - Measure P37a: % active patients with renal disease and had their BP recorded in previous 12 months
Indicator P37: Renal disease - monitoring renal disease - Feasibility - Measure P37b: % active patients with renal disease and had their eGFR recorded in previous 12 months
Indicator P37: Renal disease - monitoring renal disease - Feasibility - Measure P37c: % active patients with renal disease and had their urine ACR recorded in previous 12 months
Indicator P37: Renal disease - monitoring renal disease - Feasibility - Measure P37d: % active patients with renal disease and had their chronic kidney disease stage recorded
Indicator O38: Renal disease - dialysis - Feasibility - Measure O38a: % active patients with renal disease on dialysis
Indicator S39: Mental health - known prevalence of mental health conditions - Feasibility - Measure S39a: % active patients with mental health conditions within each mental health category
Indicator S40: Mental health - known prevalence of co-morbidity - Feasibility - Measure S40a: % active patients with mental health and also diagnosed with each of following: diabetes, CVD, respiratory and renal disease
Indicator P41: Mental health - treatment planning - Feasibility - Measure P41a: % active patients with mental health with a GP mental health treatment plan (such as MBS item number 2715) in previous 12 months
Measure P42a: % active patients ≥15 years with a BMI recorded who have weight classification (obese, overweight, healthy, underweight) in previous 12 months
Measure P42b: % active patients ≥15 years with a smoking status recorded/ updated (current, ex-smoker, never smoked) in previous 24 months
Indicator P42: Mental health - management of patients with a mental health diagnosis documented - Feasibility - Measure P42c: % active patients ≥15 years with alcohol consumption status recorded in previous 24 months
Measure P42d: % active patients with follow-up GP visit within 7-30 days of hospital discharge related to psychiatric condition
Indicator S43: Advance care planning - Feasibility - Measure S43a: % active patients ≥75 years with discussions about advance care planning recorded on file
Indicator P44: Advance care planning - Feasibility - Measure P44a: % active patients ≥75 years with advance care plan uploaded to My Health Record
Indicator S45: Safe prescribing of opioids and benzodiazepines - Feasibility - Measure S45a: Practice has a policy on the safe prescription of opioids and BZDs

Indicator S46: Safe prescribing of opioids and benzodiazepines - Feasibility - Measure P46a: Practice has a policy on discussing safe prescription of opioids and BZDs with all new prescribers
Indicator O47: Safe prescribing of opioids and benzodiazepines - Feasibility - Measure O47a: % acute patients prescribed opioids who had discussion of risk of opioid use with prescriber
**ATTRIBUTE 2**
Indicator S48: Practice goal/mission - Feasibility - Measure S48a: Defined practice mission/goal
Indicator S48: Practice goal/mission - Feasibility - Measure S48b: Mission/goal accessible to staff
Indicator S48: Practice goal/mission - Feasibility - Measure S48c: Mission/goal accessible to patients
Indicator S49: Practice profile - Feasibility - Measure S49a: Total number of staff in each professional
Indicator S50: Data sharing with local hospitals - Feasibility - Measure S50a: Able to receive electronic
Indicator S50: Data sharing with local hospitals - Feasibility - Measure S50b: Able to receive data in real time e.g. shared EHR or real time electronic shared care plan
Indicator S51: Data sharing with other health care providers - Feasibility - Measure S51a: Practice has a system for notifying GPs of specialist and allied health care correspondence
Indicator S52: Use of My Health Record - Feasibility - Measure S52a: % of active patients with Shared Health summaries uploaded to My Health Record
Indicator P53: Team-based care - Feasibility - Measure P53a: Regular clinical review meetings involving all
Indicator P53: Team-based care - Feasibility - Measure P53b: Assigned care teams to coordinate care for
Indicator P53: Team-based care - Feasibility - Measure P53c: Reports from each team member in patient
Indicator P54: Care planning - Feasibility - Measure P54a: % active patients with chronic disease who had a GP management plan in previous 12 months
Indicator P54: Care planning - Feasibility - Measure P54b: % active patients with chronic disease who had a medication management review (HMR) in previous 12 months
Indicator O55: GP and staff satisfaction - survey measuring GP and staff satisfaction with: - Feasibility - Measure O55a: Enjoyment of work
Indicator O55: GP and staff satisfaction - survey measuring GP and staff satisfaction with: - Feasibility - Measure O55b: Impact on local community
Indicator O55: GP and staff satisfaction - survey measuring GP and staff satisfaction with: - Feasibility -
Indicator O55: GP and staff satisfaction - survey measuring GP and staff satisfaction with: - Feasibility - Measure O55d: Income from work
Indicator O55: GP and staff satisfaction - survey measuring GP and staff satisfaction with: - Feasibility - Measure O55e: Time with patients
Indicator O55: GP and staff satisfaction - survey measuring GP and staff satisfaction with: - Feasibility - Measure O55f: Work/life balance
Indicator O56: Patient experience of continuity of care - Feasibility - Measure O56a: Patient Reported Experience Measure (PREM) questions on time taken for notification of abnormal test results
Indicator O57: Care plan engages patient - Feasibility - Measure O57a: Patient Reported Experience Measure (PREM) questions on patients reporting of experience with care planning
Indicator O57: Care plan engages patient - Feasibility - Measure O57b: Patient activation measure (PAM®)
Indicator O58: Follow-up following hospital attendance - Feasibility - Measure O58a: % of active patients reviewed following ED presentation within 7 days
Indicator O58: Follow-up following hospital attendance - Feasibility - Measure O58b: % of active patients reviewed following admission within 3 days
Indicator S59: Clinical governance systems in place - Feasibility - Measure S59a: Practice currently accredited according to RACGP or ACRRM standards
Indicator P60: Regular staff education undertaken - Feasibility - Measure P60a: Number of
Indicator P61: Assessment of learning needs - Feasibility - Measure P61a: Evidence of process for

Indicator S62: Data quality and completeness of demographic and key health data - Feasibility - Measure S62a: % active patients with date of birth recorded
Indicator S62: Data quality and completeness of demographic and key health data - Feasibility - Measure S62b: % active patients with gender recorded
Indicator S62: Data quality and completeness of demographic and key health data - Feasibility - Measure S62c: % active patients with allergy or 'nil known allergy' coded in patient records
Indicator P63: Improving the quality of our practice - Feasibility - Measure P63a: Evidence of work on data
Indicator P63: Improving the quality of our practice - Feasibility - Measure P63b: Data reports and date of
Indicator P63: Improving the quality of our practice - Feasibility - Measure P63c: Evidence of formal review
Indicator O64: Consumer satisfaction with quality of care - Feasibility - Measure O64a: Analysis of validated survey responses (PREMs)
Indicator S65: Registered for postgraduate GP training - Feasibility - Measure S65a: Accredited as training
Indicator P66: Engagement with student training - Feasibility - Measure P66a: Number of medical, nursing and allied health students undertaking placements in previous 12 months
Indicator P67: Research activity - Feasibility - Measure P67a: Evidence of engagement with research or Plan - Do - Study - Act activities
**ATTRIBUTE 3**
Indicator S68: Urgent access to care - Feasibility - Measure S68a: Provides same day appointments
Indicator S69: Access to non-face-to-face care e.g. telephone, email - Feasibility - Measure S69a: Process documented and advertised to patients for phone/ email access
Indicator S70: Patient demographics recorded - Feasibility - Measure S70a: % active patients with cultural and linguistic status recorded
Indicator S70: Patient demographics recorded - Feasibility - Measure S70b: % active patients who identify as Aboriginal and/or Torres Strait Islander
Indicator S70: Patient demographics recorded - Feasibility - Measure S70c: % active patients with Aboriginal and/or Torres Strait Islander status coded in patient records
Indicator S70: Patient demographics recorded - Feasibility - Measure S70d: % active patients ≥16 years with Australian Government health care card
Indicator S71: Meets the needs of Aboriginal and/or Torres Strait Islander patients - Feasibility - Measure S71a: Practice registered for PIP Indigenous Health Incentive
Indicator S72: Health related social needs assessed - Feasibility - Measure S72a: % active patients with screening for health-related social needs recorded
Indicator S73: Community engagement - Feasibility - Measure S73a: Practice has community/patient
Indicator P74: Provides healthcare to vulnerable communities - Feasibility - Measure P74a: Bulk billing for Australian Government health care card holders
Indicator P75: Meets the needs of CALD communities - Feasibility - Measure P75a: Provides bilingual
Indicator O76: Access to regular primary care provider (as measured in response to Patient Report... - Feasibility - Measure O76a: % active patients reporting they have a specific GP/ practice nurse/ care team
Indicator O76: Access to regular primary care provider (as measured in response to Patient Report... - Feasibility - Measure O76b: % active patients reporting difficulties obtaining care in previous 12 months
Indicator O76: Access to regular primary care provider (as measured in response to Patient Report... - Feasibility - Measure O76c: % active patients reporting same day response to phone call to GP/ nurse
Indicator O77: Access for low socioeconomic status - Feasibility - Measure O77a: Compare % active patients who are Australian Government health care card holders with % holding Australian Government
**ATTRIBUTE 4**
Indicator O78: Avoidable hospital care - Feasibility - Measure O78a: Use of linked data to measure potentially preventable hospital admissions

Indicator O79: Duplication of care - Feasibility - Measure O79a: Use of linked data to avoid duplication of pathology and radiology services

Practice staff		PHN staff							
Valid	Missing	Valid	Missing	Practice staff Mean	PHN staff Mean	Practice staff Median	PHN staff Median	Practice staff Mode	PHN staff Mode
68	0	26	0	3.5294	3.5000	4.0000	4.0000	4.00	4.00
68	0	26	0	3.3676	3.5385	3.0000	4.0000	4.00	4.00
68	0	26	0	3.4265	3.3846	3.0000	3.0000	4.00	4.00
68	0	26	0	3.12	3.08	3.00	3.00	3	3
68	0	26	0	3.44	3.35	4.00	3.00	4	4
62	6	26	0	3.39	3.38	3.00	4.00	3	4
62	6	26	0	3.26	3.31	3.00	3.00	4	4
62	6	26	0	3.65	3.54	4.00	4.00	4	4
62	6	26	0	3.48	3.42	4.00	3.00	4	4
62	6	26	0	3.10	3.08	3.00	3.00	3	3[a]
62	6	26	0	3.60	3.81	4.00	4.00	4	4
63	5	26	0	2.95	2.81	3.00	3.00	3	3
62	6	26	0	3.63	3.65	4.00	4.00	4	4
62	6	26	0	3.56	3.69	4.00	4.00	4	4
62	6	26	0	3.53	3.62	4.00	4.00	4	4
62	6	26	0	3.65	3.85	4.00	4.00	4	4
62	6	26	0	3.60	3.81	4.00	4.00	4	4
62	6	26	0	3.58	3.81	4.00	4.00	4	4
62	6	26	0	3.60	3.69	4.00	4.00	4	4
62	6	26	0	3.60	3.77	4.00	4.00	4	4
62	6	26	0	3.66	3.77	4.00	4.00	4	4
62	6	26	0	3.34	3.35	3.00	3.00	4	4
62	6	26	0	3.11	3.35	3.00	3.00	3	3
62	6	26	0	3.13	3.35	3.00	3.00	3	3
62	6	26	0	3.27	3.54	3.00	4.00	3	4
62	6	26	0	3.24	3.50	3.00	4.00	3	4
62	6	26	0	3.02	3.08	3.00	3.00	3	3

62	6	26	0	2.94	3.31	3.00	3.00	3	3
62	6	26	0	3.02	3.31	3.00	3.00	3	3
57	11	26	0	3.49	3.54	4.00	4.00	4	4
57	11	26	0	3.74	3.58	4.00	4.00	4	4
57	11	26	0	3.79	3.85	4.00	4.00	4	4
57	11	26	0	3.68	3.77	4.00	4.00	4	4
57	11	26	0	3.56	3.77	4.00	4.00	4	4
57	11	26	0	3.68	3.85	4.00	4.00	4	4
57	11	26	0	3.56	3.73	4.00	4.00	4	4
57	11	26	0	3.53	3.69	4.00	4.00	4	4
57	11	26	0	3.51	3.77	4.00	4.00	4	4
57	11	26	0	3.39	3.42	3.00	4.00	3[a]	4
57	11	26	0	3.46	3.46	4.00	4.00	4	4
57	11	26	0	3.75	3.88	4.00	4.00	4	4
57	11	26	0	3.56	3.77	4.00	4.00	4	4
57	11	26	0	3.53	3.73	4.00	4.00	4	4
57	11	26	0	3.53	3.58	4.00	4.00	4	4
57	11	26	0	3.49	3.54	4.00	4.00	4	4
57	11	26	0	3.56	3.62	4.00	4.00	4	4
55	13	26	0	3.65	3.69	4.00	4.00	4	4
55	13	26	0	3.65	3.69	4.00	4.00	4	4
55	13	26	0	3.16	3.42	3.00	4.00	3	4
55	13	26	0	3.16	3.38	3.00	4.00	3	4
55	13	26	0	3.58	3.81	4.00	4.00	4	4
55	13	26	0	3.55	3.81	4.00	4.00	4	4
55	13	26	0	3.53	3.42	4.00	4.00	4	4
55	13	26	0	3.29	3.31	3.00	3.00	4	4
55	13	26	0	3.29	3.31	3.00	4.00	3[a]	4
55	13	26	0	3.53	3.65	4.00	4.00	4	4
55	13	26	0	3.55	3.62	4.00	4.00	4	4
55	13	26	0	3.33	3.42	3.00	4.00	4	4

54	14	26	0	3.65	3.65	4.00	4.00	4	4
54	14	26	0	3.50	3.69	4.00	4.00	4	4
54	14	26	0	3.35	3.46	3.00	4.00	4	4
54	14	26	0	3.41	3.42	4.00	4.00	4	4
54	14	26	0	3.63	3.65	4.00	4.00	4	4
54	14	26	0	3.57	3.77	4.00	4.00	4	4
54	14	26	0	3.61	3.73	4.00	4.00	4	4
54	14	26	0	3.65	3.77	4.00	4.00	4	4
54	14	26	0	3.63	3.62	4.00	4.00	4	4
54	14	26	0	3.52	3.62	4.00	4.00	4	4
54	14	26	0	3.43	3.50	4.00	4.00	4	4
54	14	26	0	3.67	3.77	4.00	4.00	4	4
54	14	26	0	3.63	3.77	4.00	4.00	4	4
54	14	26	0	3.61	3.73	4.00	4.00	4	4
54	14	26	0	3.46	3.62	4.00	4.00	4	4
54	14	26	0	3.48	3.73	4.00	4.00	4	4
54	14	26	0	3.37	3.46	4.00	4.00	4	4
54	14	26	0	3.43	3.73	4.00	4.00	4	4
54	14	26	0	3.65	3.77	4.00	4.00	4	4
54	14	26	0	3.50	3.58	4.00	4.00	4	4
54	14	26	0	3.52	3.62	4.00	4.00	4	4
54	14	26	0	3.44	3.50	4.00	4.00	4	4
54	14	26	0	3.33	3.23	3.00	3.00	3	4
54	14	26	0	3.19	3.27	3.00	3.00	3	3
54	14	26	0	3.09	3.27	3.00	3.00	4	4
54	14	26	0	3.69	3.62	4.00	4.00	4	4

54	14	26	0	3.69	3.69	4.00	4.00	4	4
54	14	26	0	3.56	3.50	4.00	4.00	4	4
[redacted]									
46	0	26	0	3.48	3.77	4.00	4.00	4	4
46	0	26	0	3.61	3.73	4.00	4.00	4	4
46	0	26	0	3.30	3.50	3.00	4.00	3[b]	4
46	0	26	0	3.50	3.46	4.00	3.50	4	4
46	0	26	0	3.78	3.69	4.00	4.00	4	4
46	0	26	0	3.39	3.19	4.00	3.00	4	3
46	0	26	0	3.67	3.35	4.00	3.00	4	3
46	0	26	0	3.48	3.73	4.00	4.00	4	4
46	0	26	0	3.41	3.38	3.50	3.00	4	3
46	0	26	0	3.07	3.27	3.00	3.50	3	4
46	0	26	0	3.28	3.27	3.50	3.50	4	4
46	0	26	0	3.65	3.85	4.00	4.00	4	4
46	0	26	0	3.43	3.58	4.00	4.00	4	4
46	0	26	0	3.54	3.73	4.00	4.00	4	4
46	0	26	0	3.33	3.19	3.00	3.00	4	3
46	0	26	0	3.63	3.65	4.00	4.00	4	4
46	0	26	0	3.46	3.42	3.50	4.00	4	4
46	0	26	0	3.48	3.42	4.00	4.00	4	4
46	0	26	0	3.28	3.42	3.00	3.00	3	3
46	0	26	0	3.48	3.58	4.00	4.00	4	4
46	0	26	0	3.22	3.42	3.00	3.50	3	4
46	0	26	0	3.09	3.04	3.00	3.00	3	3
46	0	26	0	3.26	3.31	3.00	3.00	3	3
46	0	26	0	3.13	3.08	3.00	3.00	3	3
45	1	26	0	3.84	3.85	4.00	4.00	4	4
45	1	26	0	3.58	3.46	4.00	4.00	4	4
45	1	26	0	3.36	3.38	3.00	3.00	4	3

45	1	26	0	3.89	4.00	4.00	4.00	4	4
45	1	26	0	3.84	3.96	4.00	4.00	4	4
45	1	26	0	3.80	3.96	4.00	4.00	4	4
45	1	26	0	3.42	3.73	4.00	4.00	4	4
45	1	26	0	3.56	3.77	4.00	4.00	4	4
45	1	26	0	3.51	3.58	4.00	4.00	4	4
45	1	26	0	3.42	3.15	3.00	3.00	4	3
45	1	26	0	3.40	3.12	4.00	3.00	4	3
45	1	26	0	3.29	3.12	4.00	3.00	4	3
45	1	26	0	3.16	3.65	3.00	4.00	3	4
[redacted]									
45	1	26	0	3.64	3.12	4.00	3.00	4	3
45	1	26	0	3.64	3.62	4.00	4.00	4	4
45	1	26	0	3.56	3.77	4.00	4.00	4	4
45	1	26	0	3.69	3.88	4.00	4.00	4	4
45	1	26	0	3.69	3.85	4.00	4.00	4	4
45	1	26	0	3.51	3.77	4.00	4.00	4	4
45	1	26	0	3.67	3.88	4.00	4.00	4	4
45	1	26	0	3.31	3.35	3.00	3.50	3[b]	4
45	1	26	0	3.04	2.81	3.00	3.00	3[b]	3
45	1	26	0	3.49	3.62	4.00	4.00	4	4
45	1	26	0	3.40	3.27	3.00	3.00	4	3
45	1	26	0	3.42	3.42	4.00	3.50	4	4
45	1	26	0	3.22	3.35	3.00	3.00	4	3
45	1	26	0	3.40	3.19	4.00	3.00	4	4
45	1	26	0	3.31	3.15	3.00	3.00	3	3
[redacted]									
45	1	26	0	3.24	3.38	3.00	3.50	3[b]	4

45	1	26	0	3.29	3.19	3.00	3.00	4	3

Practice staff Std. Deviation	PHN staff Std. Deviation	Practice staff Range	PHN staff Range	Practice staff Skew	PHN staff Skew	Scores Practice staff 1 (irrelevant/infeasible)	Practice staff 2 (somewhat irrelevant/infeasible)
0.6101	0.583	2.00	2.00	-0.928	-0.656	0	5.9
0.7311	0.647	3.00	2.00	-1.176	-1.114	2.9	5.9
0.6537	0.637	3.00	2.00	-1.041	-0.535	1.5	4.4
0.744	0.796	3	2	-0.643	-0.143	2.9	13.2
0.720	0.689	3	2	-1.146	-0.584	1.5	8.8
0.636	0.697	3	2	-0.939	-0.703	1.6	3.2
0.788	0.788	3	3	-0.706	-1.159	1.6	16.1
0.515	0.582	2	2	-0.990	-0.820	0.0	1.6
0.620	0.643	2	2	-0.788	-0.667	0.0	6.5
0.783	0.891	3	3	-0.385	-0.526	1.6	21.0
0.586	0.402	2	1	-1.150	-1.659	0	4.8
0.851	0.634	3	2	-0.395	0.166	4.8	23.8
0.550	0.485	2	1	-1.147	-0.687	0.0	3.2
0.617	0.471	2	1	-1.118	-0.885	0.0	6.5
0.564	0.571	2	2	-0.695	-1.189	0.0	3.2
0.546	0.368	2	1	-1.234	-2.038	0.0	3.2
0.557	0.402	2	1	-0.986	-1.659	0.0	3.2
0.560	0.402	2	1	-0.909	-1.659	0.0	3.2
0.613	0.471	2	1	-1.266	-0.885	0.0	6.5
0.586	0.430	2	1	-1.150	-1.358	0.0	4.8
0.599	0.430	2	1	-1.604	-1.358	0.0	6.5
0.767	0.689	3	2	-0.896	-0.584	1.6	12.9
0.727	0.562	3	2	-0.441	-0.065	1.6	16.1
0.735	0.629	3	2	-0.465	-0.408	1.6	16.1
0.705	0.582	3	2	-0.736	-0.820	1.6	9.7
0.717	0.648	3	2	-0.673	-0.955	1.6	11.3
0.779	0.796	3	3	-0.674	-0.659	4.8	14.5

0.827	0.679	3	2	-0.416	-0.471	4.8	22.6
0.799	0.679	3	2	-0.626	-0.471	4.8	16.1
0.710	0.761	2	3	-1.053	-1.913	0.0	12.3
0.518	0.809	2	3	-1.869	-1.990	0.0	3.5
0.411	0.464	1	2	-1.459	-3.217	0.0	0.0
0.506	0.514	2	2	-1.237	-2.260	0.0	1.8
0.567	0.514	2	2	-0.857	-2.260	0.0	3.5
0.540	0.368	2	1	-1.500	-2.038	0.0	3.5
0.567	0.533	2	2	-0.857	-1.925	0.0	3.5
0.570	0.549	2	2	-0.705	-1.645	0.0	3.5
0.571	0.430	2	1	-0.632	-1.358	0.0	3.5
0.675	0.758	3	2	-1.012	-0.915	1.8	5.3
0.629	0.706	2	2	-0.723	-0.962	0.0	7.0
0.474	0.431	2	2	-1.725	-3.965	0.0	1.8
0.567	0.587	2	2	-0.857	-2.510	0.0	3.5
0.601	0.604	2	2	-0.872	-2.191	0.0	5.3
0.570	0.703	2	2	-0.705	-1.419	0.0	3.5
0.601	0.647	2	2	-0.731	-1.114	0.0	5.3
0.598	0.637	2	2	-1.023	-1.474	0.0	5.3
0.517	0.618	2	2	-1.080	-1.919	0.0	1.8
0.517	0.618	2	2	-1.080	-1.919	0.0	1.8
0.834	0.643	3	2	-0.719	-0.667	3.6	16.4
0.811	0.804	3	3	-0.745	-1.345	3.6	14.5
0.599	0.491	2	2	-1.132	-2.676	0.0	5.5
0.603	0.491	2	2	-0.969	-2.676	0.0	5.5
0.604	0.758	2	2	-0.892	-0.915	0.0	5.5
0.737	0.736	2	2	-0.526	-0.571	0.0	16.4
0.712	0.788	2	2	-0.492	-0.628	0.0	14.5
0.604	0.629	2	2	-0.892	-1.683	0.0	5.5
0.571	0.637	2	2	-0.802	-1.474	0.0	3.6
0.771	0.857	3	2	-0.899	-0.983	1.8	12.7

0.520	0.629	2	2	-1.052	-1.683	0.0	1.9
0.637	0.549	2	2	-0.910	-1.645	0.0	7.4
0.705	0.811	2	3	-0.623	-1.569	0.0	13.0
0.714	0.857	2	3	-0.790	-1.396	0.0	13.0
0.623	0.562	2	2	-1.487	-1.403	0.0	7.4
0.602	0.587	2	2	-1.104	-2.510	0.0	5.6
0.564	0.604	2	2	-1.115	-2.191	0.0	3.7
0.520	0.514	2	2	-1.052	-2.260	0.0	1.9
0.560	0.637	2	2	-1.210	-1.474	0.0	3.7
0.637	0.571	2	2	-0.983	-1.189	0.0	7.4
0.716	0.707	2	2	-0.849	-1.103	0.0	13.0
0.549	0.514	2	2	-1.418	-2.260	0.0	3.7
0.560	0.514	2	2	-1.210	-2.260	0.0	3.7
0.596	0.533	2	2	-1.281	-1.925	0.0	5.6
0.636	0.571	2	2	-0.771	-1.189	0.0	7.4
0.606	0.533	2	2	-0.718	-1.925	0.0	5.6
0.831	0.706	3	2	-1.209	-0.962	3.7	11.1
0.662	0.533	2	2	-0.732	-1.925	0.0	9.3
0.588	0.514	2	2	-1.477	-2.260	0.0	5.6
0.637	0.703	2	2	-0.910	-1.419	0.0	7.4
0.637	0.571	2	2	-0.983	-1.189	0.0	7.4
0.664	0.648	2	2	-0.795	-0.955	0.0	9.3
0.673	0.815	2	2	-0.514	-0.466	0.0	11.1
0.729	0.667	3	2	-0.607	-0.363	1.9	13.0
0.896	0.874	3	3	-0.677	-0.969	5.6	18.5
0.543	0.571	2	2	-1.531	-1.189	0.0	3.7

0.543	0.549	2	2	-1.531	-1.645	0.0	3.7
0.604	0.648	2	2	-1.021	-0.955	0.0	5.6
[redacted]							
0.658	0.430	3	1	-1.385	-1.358	2.2	2.2
0.577	0.452	2	1	-1.174	-1.105	0	4.3
0.695	0.648	2	2	-0.494	-0.955	0	13.0
0.723	0.582	2	2	-1.108	-0.500	0	13.0
0.467	0.471	2	1	-2.076	-0.885	0	2.2
0.802	0.634	3	2	-1.114	-0.166	2.2	13.0
0.519	0.562	2	2	-1.258	-0.065	0	2.2
0.623	0.533	2	2	-0.778	-1.925	0	6.5
0.652	0.496	2	1	-0.667	0.504	0	8.7
0.772	0.874	2	3	-0.114	-0.969	0	26.1
0.807	0.874	2	3	-0.570	-0.969	0	21.7
0.604	0.368	3	1	-2.202	-2.038	2.2	0.0
0.720	0.578	3	2	-1.632	-0.997	4.3	0.0
0.585	0.452	2	1	-0.871	-1.105	0	4.3
0.732	0.694	2	2	-0.603	-0.276	0	15.2
0.532	0.485	2	1	-1.017	-0.687	0	2.2
0.585	0.809	2	3	-0.520	-1.452	0	4.3
0.623	0.703	2	2	-0.778	-0.829	0	6.5
0.720	0.578	3	2	-0.860	-0.351	2.2	8.7
0.752	0.578	3	2	-1.395	-0.997	2.2	8.7
0.696	0.643	2	2	-0.324	-0.667	0	15.2
0.725	0.720	2	3	-0.134	-0.754	0	21.7
0.713	0.679	3	2	-0.815	-0.471	2.2	8.7
0.718	0.688	3	2	-0.576	-0.099	2.2	13.0
0.367	0.368	1	1	-1.967	-2.038	0	0
0.657	0.647	2	2	-1.308	-0.807	0	8.9
0.679	0.697	2	3	-0.583	-1.471	0	11.1

0.318	0.000	1	0	-2.561		0	0
0.424	0.196	2	1	-2.851	-5.099	0	2.2
0.457	0.196	2	1	-2.269	-5.099	0	2.2
0.690	0.533	3	2	-1.225	-1.925	2.2	4.4
0.659	0.430	3	1	-1.709	-1.358	2.2	2.2
0.589	0.758	2	3	-0.744	-2.076	0	4.4
0.621	0.543	2	2	-0.585	0.134	0	6.7
0.963	0.588	3	2	-1.541	-0.008	8.9	6.7
0.991	0.766	3	3	-1.210	-0.783	8.9	11.1
0.824	0.562	3	2	-0.812	-1.403	4.4	13.3
[redacted]							
0.609	0.711	2	2	-1.536	-0.171	0	6.7
0.570	0.496	2	1	-1.374	-0.504	0	4.4
0.546	0.430	2	1	-0.668	-1.358	0	2.2
0.514	0.326	2	1	-1.359	-2.558	0	2.2
0.514	0.368	2	1	-1.359	-2.038	0	2.2
0.695	0.514	3	2	-1.530	-2.260	2.2	4.4
0.674	0.326	3	1	-2.277	-2.558	2.2	4.4
0.733	0.745	3	2	-0.931	-0.680	2.2	8.9
0.852	0.749	3	3	-0.318	-0.898	2.2	26.7
0.815	0.571	3	2	-1.675	-1.189	4.4	6.7
0.654	0.778	2	3	-0.634	-1.080	0	8.9
0.657	0.703	2	3	-0.707	-1.578	0	8.9
0.795	0.629	3	2	-0.712	-0.408	2.2	15.6
0.688	0.849	2	3	-0.720	-0.820	0	11.1
0.633	0.784	2	3	-0.358	-0.825	0	8.9
[redacted]							
0.773	0.697	3	2	-0.769	-0.703	2.2	13.3

0.815	0.694	3	2	-1.116	-0.276	4.4	8.9

Practice staff 3 (Somewhat relevant/feasible)	Practice staff 4 (relevant/feasible)	PHN staff 1 (irrelevant/infeasible)	PHN staff 2 (somewhat irrelevant/infeasible)
35.3	58.8	0	3.8
42.6	48.5	0	7.7
44.1	50.0	0	7.7
52.9	30.9	0	26.9
33.8	55.9	0	11.5
50.0	45.2	0	11.5
37.1	45.2	3.8	7.7
32.3	66.1	0	3.8
38.7	54.8	0	7.7
43.5	33.9	3.8	23.1
30.6	64.5	0	0.0
42.9	28.6	0	30.8
30.6	66.1	0	0.0
30.6	62.9	0	0.0
40.3	56.5	0	3.8
29.0	67.7	0	0.0
33.9	62.9	0	0.0
35.5	61.3	0	0.0
27.4	66.1	0	0.0
30.6	64.5	0	0.0
21.0	72.6	0	0.0
35.5	50.0	0	11.5
51.6	30.6	0	3.8
50.0	32.3	0	7.7
48.4	40.3	0	3.8
48.4	38.7	0	7.7
54.8	25.8	3.8	15.4

46.8	25.8	0	11.5
51.6	27.4	0	11.5
26.3	61.4	3.8	3.8
19.3	77.2	3.8	7.7
21.1	78.9	0	3.8
28.1	70.2	0	3.8
36.8	59.6	0	3.8
24.6	71.9	0	0.0
36.8	59.6	0	3.8
40.4	56.1	0	3.8
42.1	54.4	0	0.0
45.6	47.4	0	15.4
40.4	52.6	0	11.5
21.1	77.2	0	3.8
36.8	59.6	0	7.7
36.8	57.9	0	7.7
40.4	56.1	0	11.5
40.4	54.4	0	7.7
33.3	61.4	0	7.7
30.9	67.3	0	7.7
30.9	67.3	0	7.7
40.0	40.0	0	7.7
43.6	38.2	3.8	7.7
30.9	63.6	0	3.8
34.5	60.0	0	3.8
36.4	58.2	0	15.4
38.2	45.5	0	15.4
41.8	43.6	0	19.2
36.4	58.2	0	7.7
38.2	58.2	0	7.7
36.4	49.1	0	23.1

31.5	66.7	0	7.7
35.2	57.4	0	3.8
38.9	48.1	3.8	7.7
33.3	53.7	3.8	11.5
22.2	70.4	0	3.8
31.5	63.0	0	7.7
31.5	64.8	0	7.7
31.5	66.7	0	3.8
29.6	66.7	0	7.7
33.3	59.3	0	3.8
31.5	55.6	0	11.5
25.9	70.4	0	3.8
29.6	66.7	0	3.8
27.8	66.7	0	3.8
38.9	53.7	0	3.8
40.7	53.7	0	3.8
29.6	55.6	0	11.5
38.9	51.9	0	3.8
24.1	70.4	0	3.8
35.2	57.4	0	11.5
33.3	59.3	0	3.8
37.0	53.7	0	7.7
44.4	44.4	0	23.1
50.0	35.2	0	11.5
37.0	38.9	3.8	15.4
24.1	72.2	0	3.8

24.1	72.2	0	3.8
33.3	61.1	0	7.7
[redacted]			
41.3	54.3	0	0
30.4	65.2	0	0
43.5	43.5	0	7.7
23.9	63.0	0	3.8
17.4	80.4	0	0
28.3	56.5	0	11.5
28.3	69.6	0	3.8
39.1	54.3	0	3.8
41.3	50.0	0	0
41.3	32.6	3.8	15.4
28.3	50.0	3.8	15.4
28.3	69.6	0	0
43.5	52.2	0	3.8
37.0	58.7	0	0
37.0	47.8	0	15.4
32.6	65.2	0	0
45.7	50.0	3.8	7.7
39.1	54.3	0	11.5
47.8	41.3	0	3.8
28.3	60.9	0	3.8
47.8	37.0	0	7.7
47.8	30.4	3.8	11.5
50.0	39.1	0	11.5
54.3	30.4	0	19.2
15.6	84.4	0	0
24.4	66.7	0	7.7
42.2	46.7	3.8	0

11.1	88.9	0	0
11.1	86.7	0	0
15.6	82.2	0	0
42.2	51.1	0	3.8
33.3	62.2	0	0
40.0	55.6	3.8	3.8
44.4	48.9	0	7.7
20.0	64.4	0	11.5
22.2	57.8	3.8	11.5
44.4	37.8	0	3.8
22.2	71.1	0	19.2
26.7	68.9	0	0
40.0	57.8	0	0
26.7	71.1	0	0
26.7	71.1	0	0
33.3	60.0	0	3.8
17.8	75.6	0	0
44.4	44.4	0	15.4
35.6	35.6	7.7	15.4
24.4	64.4	0	3.8
42.2	48.9	3.8	7.7
40.0	51.1	3.8	0.0
40.0	42.2	0	7.7
37.8	51.1	3.8	15.4
51.1	40.0	3.8	11.5
42.2	42.2	0	11.5

40.0	46.7	0	15.4

PHN staff 3 (Somewhat relevant/feasible)	PHN staff 4 (relevant/feasible)	Practice staff Scores 3-4 (%)	PHN staff Scores 3-4 (%)	Consensus
42.3	53.8	94.1	96.2	YES
30.8	61.5	91.2	92.3	YES
46.2	46.2	94.1	92.3	YES
38.5	34.6	83.8	73.1	YES
42.3	46.2	89.7	88.5	YES
38.5	50.0	95.2	88.5	YES
42.3	46.2	82.3	88.5	YES
38.5	57.7	98.4	96.2	YES
42.3	50.0	93.5	92.3	YES
34.6	38.5	77.4	73.1	YES
19.2	80.8	95.2	100	YES
57.7	11.5	71.4	69.2	NO
34.6	65.4	96.8	100	YES
30.8	69.2	93.5	100	YES
30.8	65.4	96.8	96.2	YES
15.4	84.6	96.8	100	YES
19.2	80.8	96.8	100	YES
19.2	80.8	96.8	100	YES
30.8	69.2	93.5	100	YES
23.1	76.9	95.2	100	YES
23.1	76.9	93.5	100	YES
42.3	46.2	85.5	88.5	YES
57.7	38.5	82.3	96.2	YES
50.0	42.3	82.3	92.3	YES
38.5	57.7	88.7	96.2	YES
34.6	57.7	87.1	92.3	YES
50.0	30.8	80.6	80.8	YES

46.2	42.3	72.6	88.5	YES
46.2	42.3	79	88.5	YES
26.9	65.4	87.7	92.3	YES
15.4	73.1	96.5	88.5	YES
7.7	88.5	100	96.2	YES
15.4	80.8	98.2	96.2	YES
15.4	80.8	96.5	96.2	YES
15.4	84.6	96.5	100	YES
19.2	76.9	96.5	96.2	YES
23.1	73.1	96.5	96.2	YES
23.1	76.9	96.5	100	YES
26.9	57.7	93	84.6	YES
30.8	57.7	93	88.5	YES
3.8	92.3	98.2	96.2	YES
7.7	84.6	96.5	92.3	YES
11.5	80.8	94.7	92.3	YES
19.2	69.2	96.5	88.5	YES
30.8	61.5	94.7	92.3	YES
23.1	69.2	94.7	92.3	YES
15.4	76.9	98.2	92.3	YES
15.4	76.9	98.2	92.3	YES
42.3	50.0	80	92.3	YES
34.6	53.8	81.8	88.5	YES
11.5	84.6	94.5	96.2	YES
11.5	84.6	94.5	96.2	YES
26.9	57.7	94.5	84.6	YES
38.5	46.2	83.6	84.6	YES
30.8	50.0	85.5	80.8	YES
19.2	73.1	94.5	92.3	YES
23.1	69.2	96.4	92.3	YES
11.5	65.4	85.5	76.9	YES

19.2	73.1	98.1	92.3	YES
23.1	73.1	92.6	96.2	YES
26.9	61.5	87	88.5	YES
23.1	61.5	87	84.6	YES
26.9	69.2	92.6	96.2	YES
7.7	84.6	94.4	92.3	YES
11.5	80.8	96.3	92.3	YES
15.4	80.8	98.1	96.2	YES
23.1	69.2	96.3	92.3	YES
30.8	65.4	92.6	96.2	YES
26.9	61.5	87	88.5	YES
15.4	80.8	96.3	96.2	YES
15.4	80.8	96.3	96.2	YES
19.2	76.9	94.4	96.2	YES
30.8	65.4	92.6	96.2	YES
19.2	76.9	94.4	96.2	YES
30.8	57.7	85.2	88.5	YES
19.2	76.9	90.7	96.2	YES
15.4	80.8	94.4	96.2	YES
19.2	69.2	92.6	88.5	YES
30.8	65.4	92.6	96.2	YES
34.6	57.7	90.7	92.3	YES
30.8	46.2	88.9	76.9	YES
50.0	38.5	85.2	88.5	YES
30.8	50.0	75.9	80.8	YES
30.8	65.4	96.3	96.2	YES

23.1	73.1	96.3	96.2	YES
34.6	57.7	94.4	92.3	YES
[redacted]				
23.1	76.9	95.7	100.0	YES
26.9	73.1	95.7	100.0	YES
34.6	57.7	87.0	92.3	YES
46.2	50.0	87.0	96.2	YES
30.8	69.2	97.8	100.0	YES
57.7	30.8	84.8	88.5	YES
57.7	38.5	97.8	96.2	YES
19.2	76.9	93.5	96.2	YES
61.5	38.5	91.3	100.0	YES
30.8	50.0	73.9	80.8	YES
30.8	50.0	78.3	80.8	YES
15.4	84.6	97.8	100.0	YES
34.6	61.5	95.7	96.2	YES
26.9	73.1	95.7	100.0	YES
50.0	34.6	84.8	84.6	YES
34.6	65.4	97.8	100.0	YES
30.8	57.7	95.7	88.5	YES
34.6	53.8	93.5	88.5	YES
50.0	46.2	89.1	96.2	YES
34.6	61.5	89.1	96.2	YES
42.3	50.0	84.8	92.3	YES
61.5	23.1	78.3	84.6	YES
46.2	42.3	89.1	88.5	YES
53.8	26.9	84.8	80.8	YES
15.4	84.6	100.0	100.0	YES
38.5	53.8	91.1	92.3	YES
50.0	46.2	88.9	96.2	YES

0	100	100.0	100.0	YES
3.8	96.2	97.8	100.0	YES
3.8	96.2	97.8	100.0	YES
19.2	76.9	93.3	96.2	YES
23.1	76.9	95.6	100.0	YES
23.1	69.2	95.6	92.3	YES
69.2	23.1	93.3	92.3	YES
65.4	23.1	84.4	88.5	YES
53.8	30.8	80.0	84.6	YES
26.9	69.2	82.2	96.2	YES
50.0	30.8	93.3	80.8	YES
38.5	61.5	95.6	100.0	YES
23.1	76.9	97.8	100.0	YES
11.5	88.5	97.8	100.0	YES
15.4	84.6	97.8	100.0	YES
15.4	80.8	93.3	96.2	YES
11.5	88.5	93.3	100.0	YES
34.6	50.0	88.9	84.6	YES
65.4	11.5	71.1	76.9	YES
30.8	65.4	88.9	96.2	YES
46.2	42.3	91.1	88.5	YES
46.2	50.0	91.1	96.2	YES
50.0	42.3	82.2	92.3	YES
38.5	42.3	88.9	80.8	YES
50.0	34.6	91.1	84.6	YES
38.5	50.0	84.4	88.5	YES

50.0	34.6	86.7	84.6	YES

Practice proportion of 3 (%)	Practice proportion of 4 (%)	PHN proportion of 3 (%)
37.5	62.5	44.0
46.8	53.2	33.3
46.9	53.1	50.0
63.2	36.9	52.6
37.7	62.3	47.8
52.5	47.4	43.5
45.1	54.9	47.8
32.8	67.2	40.0
41.4	58.7	45.8
56.3	43.8	47.4
32.2	67.8	19.2
60.0	40.0	83.4
31.7	68.3	34.6
32.8	67.3	30.8
41.7	58.3	32.0
30.0	70.0	15.4
35.0	65.0	19.2
36.7	63.3	19.2
29.3	70.7	30.8
32.2	67.8	23.1
22.4	77.6	23.1
41.5	58.5	47.8
62.7	37.2	60.0
60.8	39.2	54.2
54.6	45.5	40.0
55.6	44.4	37.5
68.0	32.0	61.9

64.4	35.5	52.2
65.3	34.7	52.2
30.0	70.0	29.2
20.0	80.0	17.4
21.1	78.9	8.0
28.6	71.5	16.0
38.2	61.8	16.0
25.5	74.5	15.4
38.2	61.8	20.0
41.8	58.2	24.0
43.6	56.4	23.1
49.0	50.9	31.8
43.4	56.6	34.8
21.4	78.6	4.0
38.2	61.8	8.3
38.9	61.1	12.5
41.8	58.2	21.7
42.6	57.4	33.3
35.2	64.8	25.0
31.5	68.5	16.7
31.5	68.5	16.7
50.0	50.0	45.8
53.3	46.7	39.1
32.7	67.3	12.0
36.6	63.5	12.0
38.5	61.6	31.8
45.7	54.4	45.5
48.9	51.0	38.1
38.5	61.6	20.8
39.6	60.4	25.0
42.5	57.4	15.0

32.1	68.0	20.8
38.0	62.0	24.0
44.7	55.3	30.4
38.3	61.7	27.3
24.0	76.0	28.0
33.3	66.7	8.3
32.7	67.3	12.5
32.1	68.0	16.0
30.8	69.2	25.0
36.0	64.0	32.0
36.2	63.9	30.4
26.9	73.1	16.0
30.8	69.2	16.0
29.4	70.6	20.0
42.0	58.0	32.0
43.2	56.9	20.0
34.8	65.2	34.8
42.9	57.2	20.0
25.5	74.5	16.0
38.0	62.0	21.7
36.0	64.0	32.0
40.8	59.2	37.5
50.0	50.0	40.0
58.7	41.3	56.5
48.8	51.2	38.1
25.0	75.0	32.0

25.0	75.0	24.0
35.3	64.7	37.5
43.2	56.8	23.1
31.8	68.2	26.9
50.0	50.0	37.5
27.5	72.5	48.0
17.8	82.2	30.8
33.3	66.7	65.2
28.9	71.1	60.0
41.9	58.1	20.0
45.2	54.8	61.5
55.9	44.1	38.1
36.1	63.9	38.1
28.9	71.1	15.4
45.5	54.5	36.0
38.6	61.4	26.9
43.6	56.4	59.1
33.3	66.7	34.6
47.7	52.3	34.8
41.9	58.1	39.1
53.7	46.3	52.0
31.7	68.3	36.0
56.4	43.6	45.8
61.1	38.9	72.7
56.1	43.9	52.2
64.1	35.9	66.7
15.6	84.4	15.4
26.8	73.2	41.7
47.5	52.5	52.0

11.1	88.9	0.0
11.4	88.6	3.8
15.9	84.1	3.8
45.2	54.8	20.0
34.9	65.1	23.1
41.9	58.1	25.0
47.6	52.4	75.0
23.7	76.3	73.9
27.8	72.2	63.6
54.1	45.9	28.0
23.8	76.2	61.9
27.9	72.1	38.5
40.9	59.1	23.1
27.3	72.7	11.5
27.3	72.7	15.4
35.7	64.3	16.0
19.0	81.0	11.5
50.0	50.0	40.9
50.0	50.0	85.0
27.5	72.5	32.0
46.3	53.7	52.2
43.9	56.1	48.0
48.6	51.4	54.2
42.5	57.5	47.6
56.1	43.9	59.1
50.0	50.0	43.5

46.2	53.8	59.1
28		27
27		27

PHN proportion of 4 (%)
56.0
66.7
50.0
47.4
52.2
56.5
52.2
60.0
54.2
52.6
80.8
16.7
65.4
69.2
68.0
84.6
80.8
80.8
69.2
76.9
76.9
52.2
40.0
45.8
60.0
62.5
38.1

47.8
47.8
70.8
82.6
92.0
84.0
84.0
84.6
80.0
76.0
76.9
68.2
65.2
96.0
91.7
87.5
78.2
66.7
75.0
83.3
83.3
54.2
60.8
88.0
88.0
68.2
54.6
61.9
79.2
75.0
85.0

79.2
76.0
69.5
72.7
72.0
91.7
87.5
84.0
75.0
68.0
69.5
84.0
84.0
80.0
68.0
80.0
65.2
80.0
84.0
78.2
68.0
62.5
60.0
43.5
61.9
68.0

76.0
62.5
76.9
73.1
62.5
52.0
69.2
34.8
40.0
80.0
38.5
61.9
61.9
84.6
64.0
73.1
40.9
65.4
65.2
60.9
48.0
64.0
54.2
27.3
47.8
33.3
84.6
58.3
48.0

100.0
96.2
96.2
80.0
76.9
75.0
25.0
26.1
36.4
72.0
38.1
61.5
76.9
88.5
84.6
84.0
88.5
59.1
15.0
68.0
47.8
52.0
45.8
52.4
40.9
56.5

40.9
