# Supplementary material for: Indicators of high-quality general practice to achieve Quality Equity and Systems Transformation in Primary Health Care (QUEST-PHC) in Australia: a Delphi consensus study
Source: PLoS One. 2025 Sep 5;20(9):e0327508. doi: 10.1371/journal.pone.0327508 (PMC12412998; doi:10.1371/journal.pone.0327508)
Supplement: S3 File — (PDF) [file pone.0327508.s003.pdf]

Supplementary file 3: Qualitative themes and the associated data.

Qualitative themes summary table

Themes	Subthemes	Sub-subthemes
**Use of QUEST PHC indicators and measures**	Reflect high-quality care	
	Drive and support quality	
	Support QI	
	Seek feedback	
	Benchmark	
	Identify gaps	
	Allocate resources	*Inform incentive funding*
	Help reflection	*Reflect on care provision and improvements*
	Help PHNs in their work with /support for practices	
	Potential patient care outcomes of the tool	*Patient-centred care*<br>*Practice-patient engagement*<br>*Accountability*<br>*Give voice to patient perspectives*<br>*Improve patient health outcomes*<br>*Empower patients*
	Potential issues and concerns	*Concerns/suggestions about specific indicators*<br>*Concerns about other items that should also be included*<br>*Some aspects of quality patient care cannot be measured*<br>*Evidence of these measures*

**Barriers to using quality indicators and measures**	Systemic barriers	*Deficiencies of the health system*<br>*Lack of time*<br>*Lack of funding*<br>*Lack of funding for accreditation/staff training/students*<br>*General practices are small businesses*<br>*Information flow and communication with external sources/communication between HCPs*<br>*Limitations of current practice processes/system*<br>*Workforce shortage/turnover*<br>*Lack of access to certain tools/resources*<br>*Issues with existing data linkages*
	GP factors	*Perception of threat*<br>*Will require monetary rewards*<br>*Practices do not see the value/benefits*
	Patient factors	*Patient acceptance*<br>*Unrealistic expectations*<br>*Understanding of the value of measures and their feedback*<br>*More willing to leave negative feedback*<br>*Need to educate and normalise patient input*
	The reality of general practice	*Measures are relevant but some are not realistic*<br>*Reality of Covid*
	Doubts about the tool - 'just ticking boxes'	
	Benefits of tool unclear	
	Concerns about how data will be used	
	Concerns about PRMs	*Need to be accepted by patients and clinicians*<br>*Only clinicians should interpret results*<br>*Training required*<br>*Confusion around PAM meaning*
	Risks of alienating/demoralising practices	
	Equity of access across different populations	*Adolescents*<br>*Elderly*

**Perceptions of suitability of the QUEST PHC tool for different populations**		*Vulnerable populations*<br>*People with low health literacy*<br>*People of non-English speaking background*
	Equity of the health system across different geographical areas	
**Suggestions on Implementation of the QUEST PHC tool**	Promotion and communication	
	Must have buy-in	
	Carrot not stick	
	Staff to be involved	*Staff/champions to be involved*<br>*Training*
	Integrate into current normal workflow or improve	*Must be easy to use*<br>*Other IT tools that could potentially compliment/feed data into QUEST PHC*
	Implement an appropriate number of indicators	
	Need a benchmarking/ standardisation component	
	Need accurate datasets	*Data quality/accuracy from clinicians*<br>*Data cleaning required*<br>*Concerns about representativeness of data collected by the tool*
	Data linkages needed	
	Need to overhaul the healthcare system	
	Need to be supported by PHNs	
	Suggestions of features for the tool	*A dashboard highlighting the measures would be useful.*<br>*Need a centralised data based / possibility cloud system*
	Evaluation of the tool	
	Make it a national tool	

Qualitative themes and the associated data

Higher order themes	Subthemes	Data
**Use of QUEST PHC indicators and measures**	Reflect high-quality care	Qualitative measures are not looked at enough and the role of engagement and trust and transparency would help practices have that conversation about what quality care means. What it requires to deliver care and hopefully frame the conversation in a shared language so everyone is speaking to the same things.<br><br>I think the indicators and measures reflect high-quality care quite well. I think for a lot of practices these goals would be suitably aspirational and achievable.<br><br>I thought that the indicators were the most important ones to measure and reflect the quality of care given at general practices. Individual factors such as the size of the practice - 1 doctor and a practice nurse doing reception also will impact the variety of nurse indicated action because of time constrain. Training for practice nurses can also include some of the aspects measures<br><br>The indicators reflect high quality care in General Practice extremely well.<br><br>The indicators and measures utilised in QUEST greatly reflect high-quality care in Australian general practice today.<br><br>It is a comprehensive and relevant list of indicators.<br><br>In my opinion the indicators and measures are an accurate reflection of the care in Australian General Practice<br>The indicators and measures reflect quality care really well<br><br>Some indicators that were assessed in earlier rounds share the same principals as some of the RACGP standards, such as the availability of information for patients in languages other than English, and improving patient care by collection and analysing patient feedback. Using 5th edition RACGP standards and other guidelines to

		determine quality improvement indicators and measures of best practice is long overdue. The QUEST PHC indicators are definitely a step in the right direction.<br><br>I think the indicators were good measures of high-quality of care. Timeliness and patient centered care should be at the forefront of general practice.<br><br>They reflect care very well<br><br>Most areas were covered that were needed<br><br>I believe you have covered all areas quite well.<br><br>i think this tool would be useful for all team member of general practice.<br>I think a tool such as this would be beneficial in General Practice for Practice Managers and General Practitioners.<br><br>a true reflection of quality care delivered while allowing for practice specific QI programs based on individual data<br><br>There was substantial questions in the previous two rounds. Certainly may have started conversations for general practices if they wanted to look at measures they may want to improve or implement<br><br>i think this tool would be useful for all team member of general practice.<br>I think a tool such as this would be beneficial in General Practice for Practice Managers and General Practitioners.<br><br>a true reflection of quality care delivered while allowing for practice specific QI programs based on individual data<br><br>There was substantial questions in the previous two rounds. Certainly may have started conversations for general practices if they wanted to look at measures they may want to improve or implement

		Can't remember to be honest. It seemed to align with PCMH models of care as statements of quality<br><br>I think the metrics were very current in referencing high quality care in Australian General practice<br><br>No GP could possibly challenge the relevance of any of these indicators. They are the evidence-based and gold standard. Many of them fit easily into a system that utilises chronic disease items and has an effective recall system.
	Drive and support quality	Again if tool is user friendly (as above) than it could very well support quality of care and especially QI activities that are now very much a process in general practice. The tool could streamline processes in general practice<br><br>Provide additional guidance on structures that assist with evidence-based care.<br><br>For continuous improvement and clinical case conferencing<br><br>To inform and support the care the practice provides<br><br>It should be used with coordination collaboration with practices individual need & situation.<br><br>As a General Practice Nurse of over 20 years of nursing experience, I personally love the idea of this tool. IF and it's a big IF, there was an incentive to utilise this tool and to also act on its findings, I think health outcomes could really improve.<br><br>Could be used in-house to drive quality practice<br><br>Can be part of quality improvement at general practice and can be beneficial looking at quality of care, rather than revenue generated, etc

		To encourage practices to aspire to higher levels of care. To generate practice driven quality improvement<br><br>If coupled with financial measures can be used to inform value based care.<br><br>any measurable research is benificial for quality fof care improvements<br><br>Quality improvement and uniformity in quality care.<br><br>Better inform quality service provision and meet the needs of people accessing care.<br><br>Help healthcare provider measure the quality of patient care<br><br>Continuous improvement in patients care<br><br>to inform improvements in care<br><br>To increase quality of care and patient satisfaction.<br><br>To improve patient care<br><br>To form the basis for continuous improvement<br><br>To improve patient flow and processes. Shared care<br><br>To streamline the patient healthcare, preventive health also to stop repetitive initiatives and policies in the health sector<br><br>QI Measures for practice to undertake, tracking of measures for better outcomes<br><br>Guide general practice on how best to design and deliver general practice services to maximise patient outcomes

		Seeking feedback/how it fits in to priorities
	Support QI	I think they (practices) would use it, to support their PIP QI incentives (noting that the data sharing with the PHN already provides practices with measures (%) of the PIP QI 10 key improvement measures<br><br>Ideally would be good to have a tool to use on a monthly basis as a performance measure. Would ideally form the basis of discussions at a practice level in QI meetings<br><br>To be able to periodically analyse quality of care and design PDSA for any improvement activities
	Seeking feedback	Patient activation and PREM aren't currently being used in general practice broadly. Would be great to see these items used in a way thats meaningful to patients and to practices. Practices struggle with gathering patient feedback, especially feedback based on anything clinical (more than just patient 'experience' of the service) and struggle even more to action it in a way that involves patients.<br><br>It just needs to be done and patients need to have more education on this<br><br>feedback to staff and doctors on patient satisfaction and allow for working towards improvement<br><br>Seeking feedback/why the tool is being implemented<br><br>How practices can learn from negative feedback<br><br>One way of getting feedback
	Benchmark	Standardization, comparability between practices.

		Help identify areas of focus and improvement along with creating benchmarks.<br><br>Would allow practices to have a baseline to work from in quality improvement for care provision. It would also be helpful if they are able to benchmark themselves against other general practices.<br><br>General practice can benchmark themselves based on other Australian general practices, be used as a baseline to inform and drive improvement activities<br><br>Upskilling and providing a benchmark<br><br>Need to be able to benchmark against other like practices.<br><br>Uniformity across the spectrum.<br><br>standardisation across PHNs<br><br>PRM's can be use to optimise treatment effectiveness and assess patient needs, or it can be used for benchmarking purposes.<br><br>Benchmarks of excellence also used and the high performing practices reviewed for systems that can offered to other practices<br><br>We need real bench marks. Accreditation speaks to standards but no evidence of demonstration of competency.<br><br>To compare the practice to others and the mean.<br><br>Evaluate care across practices and regions - identify gaps and needs.<br><br>comparing states and statistics

		Identify commonalities within areas of difference with an aim to achieve the same goals.<br><br>A formal system of analysing quality of care will make it easier to compare the information collected.<br><br>Help establish standards of care<br><br>Quality improvement and uniformity in quality care.<br><br>**COUNTER ARGUMENTS:**<br>bench marking practices against each other would not be favorable to practices
	Identify gaps	To identify practices who might need additional support<br><br>Areas of focus can be identified using the data, and PDSA cycles would allow practices to work towards improving specific areas of improvement.<br><br>I think this would be useful for Owners, practice managers and clinical staff. The tool could be used to guage where the practice is at and areas to improve on<br><br>i think such a tool would be very useful in identifying gaps in practice & areas for improvement.<br><br>To identify areas of care that require improvement<br><br>To find the gaps and increase the feasibility of measures.<br><br>Help identify areas of focus and improvement along with creating benchmarks.<br><br>I think they show there is always room for improvement even though measure are proving reasonably high quality

		To identify areas for improvement . To improve the skills of GPs and practice staff<br><br>Informing gaps and focus models for improvement<br><br>It will assist GP practice to identify services gap if there's any and then provide better quality of care accordingly, patient satisfaction will be higher too.<br><br>Faster way to identify patients or areas in health that need to be addressed<br><br>Practices could identify gaps in best practice & patients that they could recall -t to improve their health outcomes. They could use it to highlight them as a high performing practices or to drive quality & safe pt care. they could also use some measures to increase their revenue (e.g MH treatment plans).t<br><br>Easy to identify areas for potential quality improvement activities<br><br>Overall better understanding of gaps in patient care in General Practice.<br><br>Opportunities for improvement. Improve patient satisfaction<br><br>Using results of survey to assess the gaps in care. Take on as a QI activity to develop and improve the areas of weakness.<br><br>could support the identification of gaps to focus on (e.g. development of MFI projects or collaborations/education) - could be more flexible for practices to choose the area of interest. support in understanding general practice in their catchment & contribute to primary care teams needs assessments<br><br>Systematic reviews of data would uncover areas of improvement for individual patients, or practice as a whole.<br><br>Hopefully used by GPs as a guide and to instigate change.

		Increase awareness of where improvements to overall service need to be delivered.<br><br>Enable identification of patients groups who are not getting the care they need<br><br>upskilling of clinical staff
	Allocate resources	Resources could be directed in the area where the individual practice is found to be lacking.<br><br>Optimize use of resources and funding. Outcome-oriented measurements.<br><br>**Inform incentive funding**<br><br>Support the case for increased remuneration. Support investment in (their) practice workforce and infrastructure<br><br>Could be used by accreditors Could be used by Medicare to index funding eg for Health Homes<br><br>Confirmation of improved outcomes for patients and identifying areas where remuneration does not reflect care.<br><br>Could be used as outcome measures under an incentive program.<br><br>Identifying areas where remuneration does not reflect care.<br><br>Funding models would be different into how that influences care.<br><br>Possible funding could be provided when identified concerns are acknowledged<br><br>Incentivization as part of Practice Incentive Program possibly.

		Incentives, financial assistance to increase number of practice staff to support QI<br><br>incentives e.g. CDM points<br><br>Better funding GPs to deliver complex care is the highest priority. Better access to clinical supports such as psychiatry and specialist services. Encouraging more GPs into a declining and aging workforce through better remuneration and other incentives<br><br>Incentives get doctors and nurses more involved!<br><br>Changing incentive payments for GPs to show evidence of working towards improving through undertaking QI activities<br><br>Outcome based incentives
	Help reflection	**Reflect on care provision and improvements**<br><br>Data should be made available to all staff who could be guided in reflection on them to help effect positive change<br><br>provide goals and consistent opportunities for improvement. tie into PIPQI<br><br>Would allow reflection of own practice against peers which almost impossible for most GPs / practices for many indicators<br><br>a good set of indicators for reflection<br><br>Noticeable reminder of what is important, will evaluate care and assist in keeping it to a high standard<br><br>Promote awareness in practices, allow for QI to be implemented and processes reevaluated

		Can be used by GPs, GP registrars and supervisors and practice managers/nurses, to reflect on care provided and ways to improve this objectively<br><br>I would like to use it as an engagement tool with practices to establish how they see their practice
	Help PHNs in their work with /support for practices	i would like to think this tool could provide PHN with a way to also tier their general practices to enable phn's to guide the quality of general practice and what ways we can support them better in the areas improve quality care<br><br>It would be a useful tool for PMs,GPs,Nurses enabling and assisting with the work and support offered by PHN<br><br>Not a challenge but if the tool did support general practice in the suggested measures then outcomes could be identified for how PHN may be able to support practice and ultimately patients<br><br>supporting practices identify their interests & what they would like to focus on for QI. there are so many improvements measures - they must be of interest of the practice challenges in providing a tool without the support of a range of activities that could address indicators<br><br>Getting feedback on the tool from practices/explaining the why/how it benefits the practice<br><br>PHNs would be able to determine which practices need support and which can be moved on to the next step in their patient centered care journey
	Potential patient care outcomes of the tool	**Patient-centred care**<br><br>General practice needs to evolve at a faster pace in implementing models of care that are truly patient centered resulting in improved patient experiences and outcomes.

		Currently many outcomes are tied to monetary incentives. As a sector no regulatory governance only through independent organisations.<br><br>The measures are all relevant when we discuss Multidisciplinary Care and need to be feasible/achievable as they are relevant to ensure we have patient/person centre care and not just relying on the GP workforce buy the team that provides care. On another note we talk of integration what does this exactly mean as everyone has different interpretation particular primary care and secondary care<br><br>I think the indicators were good measures of high-quality of care. Timeliness and patient centered care should be at the forefront of general practice.<br><br>Moving toward a patient centered care model<br><br>improving the continuity of patient care<br><br>Better care that is more person centered To demonstrate the value of general practice in preventing ED and hospital admissions<br><br>Putting the focus of care back on the patient experience<br><br>increased patient centred care; increased individualised patient care; increase in ability to self-manage; ability to assess collaborative care goals<br><br>Enhancing person centred care experience based upon feedback and changing the ways of which we deliver services<br><br>To improve person centred care and wrap around services<br><br>focus awareness on their experience during care.<br><br>It would enhance patient experience, and improve quality of practice

		Promote greater focus on needs of the patient<br><br>It could be used to evaluate patient needs.<br><br>That patients would get individualised care that they are asking for<br><br>better outcomes for patients<br><br>Targeted to the needs of the patient profile of the practice.<br><br>***Team-based care***<br><br>The entire practice team for team based led care<br><br>**Practice-patient engagement**<br><br>To achieve a good caring practice all the above measures are important<br><br>considered as very important to have patient - team relationship in order to provide the best possible care to the patient.<br><br>It will increase patient-doctor engagement by providing an extra channel of communication.<br><br>Improve the quality of care Improve communications between patient and heath provider<br><br>Patients need to feel comfortable and welcomed by staff. Staff need to have the confidence of the patients

		Increased confidence and trust in the practice if it is seen to make changes in response to the PRM<br><br>**Accountability**<br><br>The more transparency there is around subjects such as prescription safety then the more accountability is shared and this protects both provider and patient<br><br>Identifies the importance of outcomes and not just token processes. Increases accountability and also team work.<br><br>**Give voice to patient perspectives**<br><br>It is a great idea to include such a tool to measure high-quality care in Australian general practice. It could be used a part of PROMs and PREMs and could be used by healthcare providers and patients.<br><br>patient perspective might not always be in line with test results, important to understand patient perspective<br><br>Give us an indication of what is important to the patient<br><br>Benefits are very much a true reflection of patient experience<br><br>Incorporating patient perspectives and change workflows<br><br>Insight into patient experience.<br><br>feeling of being included and seeing results

		Hearing from patients perspective. Empower patients on their healthcare journey. For practitioners to really understand what is important to the patient and their goals to in the end reach the clinical goals<br><br>Places pt in centre and gives the patient a voice . Also clinician accountability and a better informed approach to transforming General practice<br><br>involving patients to be part of their healthcare<br><br>PRMs should be used to create a more involvement from the patients within the practice.<br><br>The benefits of using PRMs to measure quality care would include promoting active involvement from the patients and can in improve the quality of care received.<br><br>-able to get response/data that most related to patient's experience in the care they received.<br><br>I think it gives better understanding of patient experience<br><br>Useful to get the patients feedback as they're experiencing the care from practitioners.<br><br>**Improve patient health outcomes**<br><br>Hopefully, it would prompt further assessment, follow up and patient education/self-management leading to long-term improved health outcomes<br><br>The benefits of using such a tool would assist General Practitioners with keeping a complete record of the patients health and would assist in further investigations.<br><br>Improve outcomes

		**Empower patients**<br>Enhanced health literacy and corresponding increase in patient expectations in their health outcomes (through improved healthcare in primary setting). Potential for improved choices in lifestyle behaviour, adoption of health preventative measures and earlier diagnosis and treatment of existing illness as health literacy improves<br><br>Would increase patient personal responsibility
	Potential issues and concerns	I don't recall any discussion on the pressure GP's experiencing in their workplace from demanding Patients, busy workplaces, pressures from Medicare.<br><br><br>**Concerns/suggestions about specific indicators**<br><br>From memory,, most do reflect high-quality care but some do not e.g number of adverse effects to childhood immunisations as it is an unrelated variable<br><br>I like the patient-team relationship term but not in conjunction with "validated survey tool".<br><br>Measure P53c is unclear.<br><br>Spirometry often infeasible to have accurate data when spirometer does not link to clinical management system.<br><br>Similar comment about CV risk scores and DM Risk scores and BMI calculation recorded. If the source data has been collected, then these can be calculated automatically by software. Thus measurement no longer correlates with action or outcome. May end up being a legacy measure.<br><br>Also mental health plans and other process based measurements are not outcomes, better to measure outcomes including symptom scores, PREMs and PROMs

		Measure 078a: The Lumos project with NSW Health does linkages for preventable hospital admissions. Measure 079a: Duplications can be reduced by checking a persons My Health Record and also through ePathology/eRadiology.<br><br>Often due to staffing constraints and demands of the pts there it is not always possible to get pt's phoned back on the day or in 24 hours<br><br>Measure 6b - for babies & infants - it is uncommon for general practice to have baby scales or access to baby scales in the practice.<br><br>Indicator O12 will require practices to have access to developed tools<br><br>Again really relevant but in reality mot practice have at best a generic only updated at accreditation policy. It too hard for most and they will simply put up signs saying this practice will not prescribe......<br><br>Indicator 058: reviewing is dependent on 1) the GP receiving notifications of presentation/admission into ED 2) Clear instructions provided by the hospital on seeing the GP within a timeframe 3)Patient mobility and access to their GP after their presentation/admission<br><br>Indicator 058 is only possible if general practices are able to receive timely and informative discharge summaries from hospital.<br><br>Non GP specialists need to be encouraged to cc GP specialists into results. Also Non GP specialist need to be encouraged to write to the primary care provider afer each review (incl the psychiatrists) as we dont have ESP and it takes time for our staff to follow up what is happening for our patients (and to be legislslatively annoying, if we have referred a letter is a medicare requirement).<br><br>Spirometry a little more difficult to obtain in GP settings due to COVID

		They do well to reflect high-quaility care - i think there are some measures that seem a bit tokenistic such as BMI, weight & height (weight & height certainly important for peadiatrics but i think over 18s it has less relevance).<br><br>Formal Health checks are useful in theory but too easily games as another process measure without truly leading to outcomes. Better to measure the outcome rather than the process Similar comment for O12<br>Similarly Disease Registers are going to become an old concept in a new world.<br>IT allows us to transcend disease registers and move to a new type of outreach based on different modes of stratification<br><br>These P6: Risk factors recorded are relevant, still amazes me that many clinician find it difficult to have these conversations with their patients.<br><br>Measure p44a - not all patients have a MHR - including refugees or other pt cohorts that may be larger cohorts from some practices.<br>Remote NSW experiences challenges with access to multidisciplinary team care services. Reasons include whether the service providers are available at a local level or whether people need to travel extensive distance to access allied/medical services in a regional town/city.<br><br>More work needs to be done on advance care planning and building this into usual care<br><br>Measure P8a - feasibility dependent on how results of NBCSP are uploaded to practice software & coded in practice software correctly.<br><br>***Concerns about other items that should also be included***<br><br>An additional indicator for consideration is family violence<br><br>Other measures that impact on physical and mental well being might include (with consumer input on priorities and desire for engagement): -Sleep quality/ duration -

		Social integration and contentedness -Coping strategies, building confidence and resilience -Enjoyable activities and taking small steps to boost mood<br><br>Health data would need to be designed to capture discussion between prescriber and customer on opiod risk - I am not aware this is a field in medical record although this is feasible to implement and may lead to discussions that may not have occurred otherwise.<br><br>DFV/Family violence<br><br>Mental health services/screening<br><br>Engaged leadership<br><br>Early intervention measures<br><br>Evidence of antimicrobial stewardship<br><br>Competency in skin procedures<br><br>Wound healing outcomes<br><br>Patient experience<br><br>Prevented suicide<br><br>Timely diagnosis<br><br>Comfort of feeling that there is someone who cares<br><br>early intervention measures particularly around pediatrics, e.g. mental health screening, & use of growth charts

		does not take geopolitical aspects in delivering care city versus rural<br><br>Modestly. There are many factors that these don't capture.<br><br>The indicators definitely reflect high quality care, the only thing missing will be patient experience.<br><br>Very comprehensive suite. Need to cone down the measures. The other measure to look at is financial sustainability of the practice<br><br>Evidence of anti-microbial stewardship. Determining GP competency in relation to undertaking various types of skin procedures. Wound healing outcomes especially concerning chronic wounds.<br><br>***Some aspects of quality patient care cannot be measured***<br><br>Patients want you to be there for them and you cannot underestimate the value of the relationship when you create a safe non-judgmental space for a patients to guide them through their health journey. Not sure how you measure that? They are generally bemused by surveys!<br><br>I believe the hallmark of excellence in general practice is to provide a safe and non-judgemental space with a healthcare provider team that they know and trust and where their personalised health outcomes are paramount at each consultation. It is hard to know how you measure this type of relationship with a tool. It is my experience that over 33 years this type of care is becoming more and more difficult for a patient to access.<br><br>Sadly not always is information specific to patient documented so that it can be mapped but this does not mean that there is not high-quality care given in general practice.

		They are good indicators for guidance to inform quality care. However, nuances of practice functions and clinical delivery may not necessarily translate to the patient and outcomes and so can be a guide but may not really indicate a high performing practice<br><br>The unmeasurable value that patients cannot articulate in a survey of what it means to ahve an advocate they trust in the health system. How do you measure a prevented suicide, a timely diagnosis and the comfort of feeling that their is someone who acres what happens to you in all ways and not just to meet criteria. It si not that the indicators are not fantastic it is jut that they cannot take time and focus away from the unmeasurable !!!!!!<br><br>Sadly not always is information specific to patient documented so that it can be mapped but this does not mean that there is not high-quality care given in general practice.<br><br>Quality of care is different for each person so still think this will be very hard to gauge<br><br>rigid, always open minded that some patients are out of the norm but have conditions<br><br>Some aspects of care cannot be measured by a tool and this may tend to under rate certain aspects of care<br><br>one size might not fit all -<br><br>Ideally yes however need to be careful as the tool would be reflecting the overall general practice, some practices may score low but have GPs that provide a high level of individual care. Its a hard shift for some to see that overall service to patients includes both the clinical care and the access, records, data analysis and other systems and processes in place in the practice<br><br>**The evidence of these measures**

		It’d be nice to see evidence for these measures and change in practice / better outcomes from overseas experience
**Barriers**	Systemic barriers	**Deficiencies of the health system**<br>These (measures) are a necessary want and need to manage the complex, chronic health needs of patients that compound overtime but there is a significant lag time in the investment of money, people and resources to manifest the requirements. The KPI's being discussed are more theoretical and ideological and does not align with a funding model via MBS. MBS significantly undervalues large portions of the health sector and will not deliver on provider and patient satisfaction, it does not understand what is needed and how measure and appropriately remunerate complex, chronic care. In reality it seems tokenistic, short sighted and ultimately is used as means to validate a user pays system rather than champion the integrity of what universal health care can represent. There is also a lack of inclusion with patients on the enormity of what is being asked and engaging them in the responsibility of how they access a publicly funded health system and their role in supporting sustainability and realigning expectations. The sell is always mightier than the follow through and the money not often going to where it matters but rather less translational and more data, policy and manager driven with swathes of money going to the wrongs parts. Counter to this there is punitive targeting of Primary care that is disengaging rather than encouraging the right directional movements for a sustainable health system. Its fracturing team based care. It is divisive and pitting different disciplines against each other rather than fostering cohesion.<br><br>Barriers: - access to specified team-member/provider within suggested timeframe - PAM is a costly/lengthy process to integrate within practice - My Health Record currently not used to maximum and not accessible by all team members - inability for 'shared care plan' due to requirement of additional practice software - % of f/up post post ED presentation/hospital admission,, will not account for patients not attending for reviews as recommended<br><br>Advance care plans fit well with Health Assessments but unless it has changed you couldn't upload to MHR.

		The problem is that patients do not always want to come in for HA s and M plans. They are best received when done opportunistically. Prior to 2014 our system achieved the best outcomes and doing these items when the patient chose to come in for something else was very very "patient-centred". Changed to medicare have meant that you cannot do them on the same day and this was to my mind one of the greatest blows to patient-centred care. General practice is on its knees financially if you add any more hoops for it to jump through to receive the same funding you will see the collapse in general practice care.
		**Lack of time**<br><br>Documenting vaccinations is feasible if done in the practice. Documenting vaccinations done elsewhere can be time consuming and could be done if the practitioner has time available<br><br>While the measures are relevant,. It is also time /resource consuming to report on a large number of measures.<br>All important. Comes down to time and in what consult you do thse. Some I update like meds lifestyle, some are can of worm questions and will nee more time. We really need access to a yearly health check medicare no for all patients. Also having time to do this can be tricky. The concept is good - have to consider how we implement it in a practical sense.<br><br>A lot of time needs to be spent collecting this information and while I think it most of it highly relevant I find access to required services can be difficult to access and often have such long waiting times that most patients who would benefit just wont or cant spend the time and effort. I feel often its a tick box for those higher up so they can say yes my clinic, suburb, region (what ever) offers Blah blah.. but often to little too late and see above comment

		How to ensure these/any measures/indicators will be given protected time to be collected, collated, and applied. All great in theory but staff need time for these to be assessed and documented to be meaningful/useful.<br><br>A great adjunct to care but again don't allow it to be used to demoralise, devalue and consume valuable time. We already have very few graduates keen to work in general practice<br><br>Some providers would be hesitant to utilise it due to time and workforce constraints.<br><br>GPs hesitancy and lack of time for nurses and doctors alike<br><br>time to spare for this to be done<br><br>Time and resoruces to complete. Poor understanding of of the indicators.<br><br>lack of time and resource to implement and evaluate outcomes.<br><br>Finding time to do it and implementing changes to benefit patient and staff<br><br>The time and cost of implementing.<br><br>Engagement and regular use of the tool (can be difficult for practices to find the time or see benefits of using tool regularly)<br><br>quarantining time to using the tool - this is a HUGE ONE data cleaning to reflect true measures the data extract recipes from 3rd party data extraction tools difficulties of the tool taking into consideration general practice a small business<br>finding a phn representative that has time and enthusiasm to follow through<br><br>Challenge will be that your GP continually wants to ask about 100 things to tick boxes and all you want to do is see them about your medical condition and you cant get in for a week.

		Yet more hoops to jump through...Incentives<br><br>Funding - already stretched staff time poor<br><br>Protected time for each practice<br><br>change agents that do the work with the practice free of charge. I currently have a practice facilitator that I enjoy engaging and talking about delivering quality care but after there is neither the time, the man power, the resource that seamlessly integrate to deliver on the sell. Rather creates more " WORK"<br><br>Time (for Patients and GPs) and cost to the practice with a lot of competing demands. Need to address privacy concerns etc.<br><br>Most practices would be much to busy to implement this<br><br>All worked well just feel it might be a moot point as I don't know it will accomplish much in the present ridiculously busy environment of General Practice<br><br>Not enough data could be collected due to time constraints or other barriers faced by participants.<br><br><br>**Lack of funding**<br><br>Patient activation measures are often not feasible due to the cost involved to have access to the tool.<br><br>I'm loving these measures. As an experienced Chronic Disease Nurse of over 2o years this would be my ambition for all people with Chronic Disease. However the lack of specified funding (the only way to attract GPs to assess, record and follow this information) mean it isn't often a priority for busy GPs. There needs to be funding that

		is SPECIFIC to collecting and acting on this information. Failing GP engagement why not allocate and dictate funding to utilise the already available work force of General Practice Nurses. This pool of professionals is already in place through all of Australia. passionate, qualified and willing to up take this area of Health Promotion and Disease Prevention. But they are still underpaid anywhere up to $15 PER HR!!!!! when compared to their equivalent in public ACUTE care and usually treated as a tool to increase GPs earnings rather than the valuable, perfectly placed resource for Chronic Disease Management they really are.. Once someone in a position to organise this listens to this advice and acts on it I am convinced the health of the general population will improve dramatically and for the "bean counters" acute health care costs will also dramatically decrease!!<br><br>Nice idea but never supported by Medicare or any health administration!<br><br>See previous comments. Measures in which I have ticked feasible instead of somewhat are the ones where funding is available under MBS item 721/23 732 x2, and 2715 and under some measure of control/follow up by he GPN. Some items where I have chosen somewhat relevant instead of relevant are because I would record then under the previous category for measurement/follow up any way. For example weight in someone on antipsychotics, kidney health or BP in someone already being actively if its happening (usually by GPN for diabetes)<br><br>COVID has been huge for us in our small community. We are doing our best however lack of renumeration for nurses and doctores form medicare makes it difficult to provide the level of service we would like. We cannot afford to essentially work for free anymore.<br><br>Some questionnaires are commercial - will need to cover costs<br><br>It is frustrating answering the questions without being able to describe the barriers to feasibility. In the back of my mind, all the time is which of these indicators are going to be forced on general practice. I believe in the ideal of universal health care! So yes all

		healthcare cardholders should be bulk-billed but with the medicare freeze and not allowing a management plan on the same day as a consultation has meant that in order to remain open we have to charge. We even have to charge healthcare cardholders once a year. In addition in a small practice every time you add another survey and analysis of data you add additional cost to the practice.<br><br>We provide a high quality service however BB is not tenable in the long term for most of our patients/. Mental health care also needs to be reviewed as this is very time intensive and poorly renumerated for GP's who provide a lot of support for those who cannot access psychologist or psychiatrist. The same could be send for perinatal neurodiversity care and child and adolescent mental health<br><br><br>This is a great start but put funding DIRECTLY and SPECIFICALLY into funding primary practice nurses and you will get your data and improved health outcomes<br><br>Even if you can get staff to make time to utilise the tool it will not translate into high-quality care until there is funding available to put measures in place to rectify any shortfall the tool identifies<br><br>Support investment in their practice workforce and infrastructure<br><br>I think most of them would be great from a medical/nursing perspective but in the current funding models of Primary Care thy re a bit idealistic!<br><br>Relatively well. Most reflect standard practice, some reflect ideal practice that we know is not routinely followed for a number of reasons mainly around time and funding constraints and some are aspirational/not achievable in the current way general practice works<br><br>Implementation would be challenging; will there be incentives for practices with already limited time especially with continued Covid-19 demanding their time from a preventative (immunisation) and treatment management view. Ideas to support

		uptake such as general practice incentives to enhance business model, invite practices to participate in pilot before wider roll-out, training opportunities etc may influence participation<br><br>What additional resources would be made available to support areas of concern identified through the tool?<br><br>incentive to fill out questionnaires and to look at results<br><br>Funding that is payable on when directly related to and supported by evidence that this tool is being used and findings acted upon. Funding that would probably best be paid to allow for nurse time to apply the tool and (run nurse run clinics perhaps) to address the shortfalls. Nurses could identify using the tool, recall patients, address basic assessment, collate information and prepare paperwork for Doctors to complete.<br><br>Funding that remains a carrot and is not ten a stick!<br><br>that is payable on when directly related to and supported by evidence that this tool is being used and findings acted upon. Funding that would probably best be paid to allow for nurse time to apply the tool and (run nurse run clinics perhaps) to address the shortfalls. Nurses could identify using the tool, recall patients, address basic assessment, collate information and prepare paperwork for Doctors to complete.<br><br>MBS funding and policy change to support<br><br>Buy in with GPs, funding, training, and implementation supports.<br><br>Incentives, and or funding to support protected time. Capacity building and resources, tools to support data collection on associated measures<br><br>Education, increase of funding to implement<br><br>Specific PHN funding and dedicated / upskilled staff

		Provide training, funding<br><br>Funding as there will be extra work Resources Mandated as part of PHNs work<br><br>Commonwealth funding to expand programs;<br><br>Become part of routine performance indicators, financing to support practices in implementation<br><br>Funding - we already assist in the implementation of a lot of digital health initiatives and technologies<br><br>Funding to support the roll out of this and access to the data<br><br>At all costs it must be PROTECTED time and SPECIFIED funding, without accountability or direction, funding will just disappear without delivering any of the potential benefits to the individual patient and immediate and future savings for the health care budget.<br><br>More funds and a national e -system<br><br>Like "myschoo"l there needs be a socioeconomic index and practices should be subsidised in that manner and services to raise health equity based on a whole of practice assessment contextualised by its location.<br><br>Funding, Training, Mentoring and Support for PHN's and General Practice Teams<br><br>Lobbying for better funding so we don't have to charge patients and this means universal access.<br><br>Focus on quality improvement Funding Upskilling of staff Communicate the benefits Make it easy

		Provide resources, tools, funding and incentive to general practice. identify and improve workforce issues, increase mbs and create more flexible MBS item numbers for preventative care<br><br>Adequate funding for primary healthcare so that we can have enough staff to provide the high quality of care that we strive to provide.<br><br>Increase of funding and education for clinical staff<br><br>***Lack of funding for accreditation/staff training/students***<br><br>Existing practice workforce can decrease practice capacity for GP registrars and medical, nursing students. Allied Health Student placements rarely seen in general practice unless the practice employs the allied health provider. Currently there is no financial remuneration for placement of nursing students or allied health students in general practice, only medical students<br><br>While practices are not held accountable a lot of training of staff depends on the integrity, interest and capability of the individual staff member. While there is no PROTECTED and FUNDED time for these activities much as I would like and support the idea it probably wont happen in reality<br><br>Accreditation, training and access to students are not all feasible (and sometimes not relevant) in some primary care settings, especially enhanced primary care settings (e.g. headspace). Part time GP workforce and lack of funding for practice and clinical supports are challenges.<br><br>**General practices are small businesses**<br>Time and renumeration is the biggest barrier currently. Any time for the other comes at cost from income generation in the small business that is General practice and the

		smaller your practice the more individual compromise that comes with when running a bulk bill practice that is still pricing what we do at costs form the 80's<br><br>I think the measures are good - but it's important for measures to consider that practices are small businesses & it's not uncommon for practices to want to meet measures but where the bottom dollar is a barrier to time & effort.<br><br>although I think that would be quite unrealistic especially the feasibility in my current situation as solo practice owner<br><br>this is a HUGE ONE data cleaning to reflect true measures the data extract recipes from 3rd party data extraction tools difficulties of the tool taking into consideration general practice a small business finding a phn representative that has time and enthusiasm to follow through<br><br><br>**Information flow and communication with external sources/communication between HCPs**<br><br>We are still do so many phone consults, especially of patient of another practice to which doctors have remote access. So, patient are generated as a "new patient" but the patient don't visit practice and the doctors don't complete the patient file with all the relevant information like social history & so on.<br><br>Currently its the lack of interoperability and easy access to shared information that is the issue. If data can be easily merged and uploaded and shared between different providers there is greater opportunity for catchment of this information. Otherwise there has to be very good systems and deliberate processes that make what seems like simple requests optional, compulsory.<br><br>Again great in theory, but until hospitals for example ae compelled to ensure admission/discharge/follow up instructions are sent to the General Practice in a timely manner its all just "lip service" I can not believe in this electronic age that information

		cannot just be sent as soon as the letter/notes ae typed in. the expectation (and correctly so) is that the GP is the information centre and up to date with patients needs/conditions/medication etc but if information isn't sent sent how can they be<br><br>Not sure what is meant by linked data in terms of how this would measure potentially preventable hospital admissions, in any case feasibility will be tied to inconsistent use of data systems and linkages between primary and tertiary health. Eg inconsistent use of MHR in tertiary system.<br><br>Now hospital files are all online, previous admission notes are easy to view after hours. There is a downfall on quality discharge notes reaching Primary Health providers.<br><br>often there is IT issues and transfer of discharge summaries are slow and not done - pt's don't make appts and clinics are not notified of admission or discharge plan<br><br>Comment about Disease registers previously made - ie that this is now an older way of ti8nking when registers were paper based due to limited information management capacity and paper records. IT now allows much better refinement, dynamic registry's and more nuanced consideration of multi morbidity and impact.<br><br>While the measures are almost entirely all relevant, to actually report on all of the above measures would be resource intensive. So while almost all technically feasible, such a comprehensive review may be challenging for many practices, although I am not familiar with how some of the newer assessment and audit tools might assist in the process/ accuracy etc. Some of the feasibility issues are to do with communication (eg from hospitals and other clinics) and acknowledging that many people have care delivered across a number of clinics, even where they are active (depending on access, convenience, preference for different issues eg physical V sexual V AOD V mental health). Presuming that an active patient is not obtaining care elsewhere is problematic.<br><br>**Limitations of current practice processes/system**

		Question about feasibility depends heavily on softeare systems, coding and measurement tools. Currently much is not easy that could be easy.<br><br>All these indicators are relevant to collect as part of evidence base care as it allow to start managing these disease. But the systems for collecting this information is the challenge and why? Does it need to rely on one person who manages the patient or can this be done by a number of people that can access the information and provide a plan to the patient.<br><br>I think soft ware that automates the recipes, processes and prompts- where there are prompts obstructive to certain processes without the necessary inputs can condition practices and behaviours to inform the feasibility of outcomes of data collection. I do however believe that covid will be become obstructive to such investigations such as spirometry and where patient contact is paramount. I do believe though that patients are more ready for wearable technologies and engagement with these wearable will give healthcare teams greater access to almost "free" data<br><br>general practice software does not currently allow for data regarding "Reason for visit" to be extracted<br><br>Through 30 years of general practice, I have strived to have a practice that achieved multidisciplinary team care and interaction with the relevant health community for the care of patients. We have an ongoing goal to have a management plan for all patients with a chronic illness and I tried on many occasions to create systems for medication reviews in the practice. There are so many factors that make achieving all these goals so incredibly difficult and at times impossible. It had got harder not easier over the years! Please talk to GPs from many different practices as well as doing this research to understand why things may be relevant but not feasible.<br><br>With regards to access - telehealth and flexibility in how care is delivered and remunerated needs to be significantly reevaluated with archaic definitions of what constitutes a consult revised.

		Measure O78a - this is possible through our data linkage project called Lumos - we are already providing reports on this to our practices.<br><br>**Workforce shortage/turnover**<br><br>Many parts of rural and remote NSW experience workforce shortages; QI is often challenging for locum GPs due to short length of contracts. The tool may present additional pressures to already time-poor staff often working in areas of social disadvantage<br><br>High staff turnover would mean not all members of practice staff would understand the tool and how it is used<br><br>More time made available through additional workforce within a practice - currently short staffed in many areas. Where is the benefit for practices to adopt this tool?<br><br>Sparingly and sporadically, we do not have the workforce to be doing more and more with inadequate funding and staff leaving primary care to find hospital roles or roles outside of healthcare.<br><br>**Lack of access to certain tools/resources**<br><br>Practices currently do not have a tool to measure patient activation.<br><br>it is a very good concept though it is hard to implement at present due to lack of support from infrastructure and lack of awareness in patients<br><br>Practices functioning with a high number of locum GPs and / or agency nurses (or lack of nurse) have additional challenges in maintaining processes for continuous quality improvement; such as maintaining the medical team's knowledge of available support

		services (including linguistic services), understanding community health needs and social impacts, cultural understanding etc.<br><br>Some indicators extremely onerous to apply. Incredibly hard to add more pressure to practices we already know are way overstretched with minimal resources / funding to do this<br><br>The challenges of this tool at a GP level could include not having the adequate resources on hand.<br><br>Time and education to understand the tool<br><br>Resources to support practices. Reluctance of practices to be supported.<br><br>Could create division between PHN and practice which PHNs strive to minimise. Would be seen as additional pressure from PHN. PHN resources limited and might best be used elsewhere.<br><br>Don't have the knowledge to answer<br><br><br>**Issues with existing data linkages**<br><br>Some matched data sets such as ED and post discharge review data may not be as easily available or in a timely manner. Also frequently comes back to practice now in a deidentified format thus limiting utility<br><br>Indicator P9 - dependant on practice data collection, coding & updating of pts who receive vaccinations outside of the general practice (e.g influenza)<br><br><br>**Multiple complex factors**

		These are all feasible and relevant endeavors but need to be done with genuine intentions and with easy delivery that accounts for the fragmentation of not only services but service delivery tools and the practicalities of the time to pursue these metrics. Funding models have the most impact on informing the shape and the metrics of patient care currently. The metrics should be informed by understanding the expectations of individual and the system and health belief models that frame these expectations
	GP factors	Possibly where i have listed above somewhat feasible is that clinician feel pressured in collecting this information as it takes time and the patient may have presented for other matters. This then ends up being opportunist rather than planned care. We need to develop other systems or ways to be able to collect this information and maybe away that we present these indicators to an individual<br><br>***Perception of threat***<br>GPs might perceive it as a threat and resist Govt may use such a tool as a funding lever General practice not given the skills to undertake quality improvement<br><br>A lot of clinicians are very nervours on how to use such tools and feels that it takes away from them<br><br>***Will require monetary rewards***<br>I fear, very few practices would use this without any monetary gains. It would take precious time, there would need to be an incentive<br><br>**Practices do not see the value/benefits**<br>It will be ignored as another application to use but not touched<br><br>Non compliance, no interest from GPs and tool not completely honestly.

		Low engaged Practices will have low motivation.<br><br>Very useful but not sure that practices would see the benefits<br><br>Pushback or lack of interest from some practices<br><br>unsure staff that have the interest in this<br><br>Pushback or lack of interest from some practices<br><br>unsure staff that have the interest in this
	Patient factors	Risk of biases. Worry about saying negative things about practices / staff whey have known for years. Not knowing any different for comparison for some long-term patients.<br><br>PRMs is difficult to be implemented on patients who cannot read<br><br>Practices may try to game the results<br><br>For consideration is whether individuals have capacity and capability to engage with service providers (socio-economic disadvantage - technology, financial, transport, personal/family/community priorities, health literacy, health expectations and more)<br><br>These measures are all dependent on Pt willingness to discuss and compliance buy in<br><br>Patients compliance and not wanting to disclose information is a barrier along with family scared to discuss end of life care and advanced care directives for a person that is well<br><br>Difficulties in getting patients to attend appts particularly when they have a fear of COVID along with pt's being honest of what is happening in their lives

		Bias- patients my not be totally honest especially if they think their GP will see results<br><br>Patients not reporting truthfully of accurately. Pateints reporting too broadly.<br><br>How reliable results are, as everybody perception is different, e.g. pain tolerance<br><br>Take into consideration skewed data from upset customers or from people that are not interested in giving feedback.<br><br>**Patient acceptance**<br><br>While the measures are relevant, the feasibility question sits more with patient acceptance of being asked the additional questions.<br>While all of the above measures are relevant and most are at least somewhat feasible, there is a limit to the use of indicators that a patient will tolerate and that can be meaningfully digested, analysed and utilised. It would be more meaningful to have the consumer perspectives on these indicators given it is they who will be asked to complete surveys and be engaged around results and interventions in response etc<br><br>patients are not thinking about cost when they think a test is needed. and more often than not they feels a test is needed to confirm a diagnosis.<br><br>These measures are all dependent on Pt willingness to discuss and compliance buy in<br><br>Patients compliance and not wanting to disclose information is a barrier along with family scared to discuss end of life care and advanced care directives for a person that is well<br><br>**Unrealistic expectations**<br><br>That some patients might have unrealistic expectations, that most practices wont make any changes so its a waste of time/resources to have them

		not all patients receive the care they believe they should, especially if they have been googling, and this could result in unfavourable response<br><br>The equivalent of a bad google review from a patient with unrealistic expectations.<br><br>**Understanding of the value of measures and their feedback**<br><br>Might be difficult for some patient groups to use PRMs Suitability of measures Patients not seeing the value<br><br>Patients have to have an understanding of WHY this is important for their healthcare otherwise low uptake might be the result.<br><br>**More willing to leave negative feedback**<br><br>-might get more negative data than positive as patient tend to more willing to leave feedback/report when they are not satisfying with the services they get<br><br>**Need to educate and normalise patient input**<br><br>Often pt's have no idea that they can have input into their own care plan and need to be educated to do so<br><br>The credibility of health providers to ask these questions need to be supported and empathy and non judgment are important considerations in asking these questions but they also need to be normalised as part of a health encounter and understood in context. The teaching of a standardised approach to these lifestyle factors enquiry but so is the teaching of the expectation that your health provider and their team have a legitimate reason for enquiry is important too. And check in tools for patients that get

		them to fill in previsit and while waiting for the appointment with their doctor should be incorporated into medical software
	The reality of general practice	There has been a lack of acknowledgment of inherent biases that exist in a model of care largely still based on a biomedical model, and fee for service structure that is being asked to deliver on measures and indicators that are incompatible with each other.<br><br>I don't think the measures accurately reflect what is happening in General Practice.<br><br>If high quality care is seen to be about improving my health or curing me this will be challenging a patient with terminal illness maybe receiving high quality care and this does not mean they will be cured but some may see that because they were not cured they did not receive high quality care. The language that will be used in the toll has be be clear<br>All important. Comes down to time and in what consult you do thse. Some I update like meds lifestyle, some are can of worm questions and will nee more time. We really need access to a yearly health check medicare no for all patients. Also having time to do this can be tricky. The concept is good - have to consider how we implement it in a practical sense.<br><br>The feasibility (and therefore the relevance) for some of these measures is more challenging in enhanced primary care settings with part time workforce, e.g., same day appointments, communications, etc.<br><br>there is lag time in evidence of MBS item numbers in the EHR, there is also variability in ability to view pathology and imaging of patient test in their EHR and patient recollection is poor and patients are not thinking about cost when they think a test is needed. and more often than not they feels a test is needed to confirm a diagnosis.<br><br>We are still do so many phone consults, especially of patient of another practice to which doctors have remote access. So, patient are generated as a "new patient" but

		the patient don't visit practice and the doctors don't complete the patient file with all the relevant information like social history & so on.<br><br>Its all very difficult at the moment. dr shortage, nurse shortage, patient demands. telehealth makes measuring BMI etc extremely hard. I have had reports of a lot of patients having telephone consult while they are still performing work duties, an example of this is a builder asking a dr to hang on while he tells his client the door should be white?? lockdown has changed patient perception and expectations of health care. seems to be a lot more casual and direct health not indirect/ preventative medicine. Patients main concerns are the here and now.<br><br>this matters can be addressed if patient presents themselves at the clinic<br><br>Interview a range of Gps and actually ask them to think about how this will work intheir practiec. Theindicators are obviously great and feasible but they must be contextualised by people who understand the enormous stress in general practice at present.<br><br>The indicators and measures all reflect practices of high calibrate however not all high performing practices can focus on all of these areas at once.<br><br>lcompeting QI areas with the inability to address all at once, possibly incorrectly indicating care gapsi<br><br>Cit has the risk of being overwhelming for practices - eg, where do they prioritise their efforts. or they could question what they should be aiming for (obviously everyone should aim for 100%, but practically it may not be achievable all the time). there seems to some cross-over with some of the RACGP 5th standards, practices has many self-assessment tools to use. if there is a practice reasons that could drive uptake use of a tool then that would be helpful.<br><br>Identify what's realistic and achievable on the ground.

		Would not reflect the key differences and areas of needs for different practices.<br><br>Staff not trained in using this tool and patient has an expectation and isn't fulfilled<br><br>The tendancy for people to report the negative and not having a balanced amount of responses<br><br>Practices may try to game the results<br>Not always an overall indication of practice, but the view of 1 person<br><br>Higher expectations and not meeting the expectations of patients<br><br>Can be punitively used by users. As a provider; its hard to stay objective and its very hard to not take things personally or unhear or unsee somethings. So qualitative data also has the risk of emotive fallout that can be hard to process objectively<br><br>Can be too subjective and difficult to code and therefore objectively measure<br><br>Managing realistic expectations from both a practice, consumer and stakeholder level<br><br>Previous two rounds covered significant measures and indicators but unsure if a true understanding of general practice and their desire for best outcomes for patients and practice can be compromised by i.e. HR isssues -change of staff, lack of being able to employ staff. Then is it also acknowledged circumstances i.e. pandemics etc can also impact general practices.<br><br>General practice does not have a tool to measure PAM score - I have put infeasible for this question because of this barrier.<br><br>May not capture the local context of the general practice environment

		**Reality of Covid**<br><br>This section was difficult for me to answer. Im not clinical, im management. I believe it all to be relevant but the feasibility is questionable. lockdown, covid, nurse shortage, dr shortage, some things will not be recorded in the data in patient records. we do educate and advise but then booster shots, children vaccination for Pfizer, no notice from department just a national announcement. I know this survey is for health care in general but we need to also study the current environment and how it is impacting health care at the moment. Just an example of this was Sunday the announcement of 5 month not 6 month boosters. General Practice were not notified prior to public. We had no vaccines, no extra staff, so im sure things were overlooked ie loading health summaries. we were penalised by department of health last pip quarter because we loaded 12 not 15.<br><br>All the factors noted above are most definitely relevant but need to take into account the external factors (like the pandemic, personal situation).<br><br>Due to impacts of COVID training has decreased and staff levels decreased so unable to train medical and nursing students
	Doubts about the tool - 'just ticking a box'	Feel that this is just about ticking boxes and most of this does not reflect the measure of care at all<br><br>They do well to reflect high-quaility care - i think there are some measures that seem a bit tokenistic such as BMI, weight & height (weight & height certainly important for peadiatrics but i think over 18s it has less relevance).<br><br>As I do not think it does reflect the level of care , I think again this tool is just ticking boxes<br><br>That it ticks a box rather than actually translates to practice change and patient outcomes by supporting the quadruple aim

		That it's not focused on care rather than just filling in requirements<br><br>Feel it's all about ticking boxes and more of a waste of time than achieving anything worthwhile<br><br>Comments on feasibility relate to the system as it currently stands with fragmented information and patient mobility without a defined patient cohort (ie registration). This changes should there be registration and better data sharing across the system. Others this is going to require extra staff and risks becoming **a tick box exercise** where it is gamed by employing staff to comb databases.<br><br>These risk factors are only relevant if they are part of appropriate screening and the patient has wherewithal to make changes. Otherwise these will become another pointless box ticking exercise.<br><br>I wonder who will take the time to use the tool, and if it is used who will address the shortfalls identified, recall the patients and implement changes?????<br><br>A lot of time needs to be spent collecting this information and while I think it most of it highly relevant I find access to required services can be difficult to access and often have such long waiting times that most patients who would benefit just wont or cant spend the time and effort. I feel **often its a tick box for those higher up** so they can say yes my clinic, suburb, region (what ever) offers Blah blah.. but often to little too late and see above comment<br><br>In a perfect world, we could tick the box that all these measures have been addressed and the best outcomes achieved. Where there is extensive external campaigning and promotion such as cervical screening, flu vaccines etc it is easy to streamline these in practice systems and achieve good outcomes and patients are very willing and compliant in most cases. With more sensitive measures such as weight, alcohol use, sexual practices etc there needs to be a more nuanced approach where you often pick your moment's in the safe space of general practice within a therapeutic relationship

		to make a real difference. Ticking a box that you have had a discussion is a very simplistic approach.<br><br>Need to explain to patients why there is time to ask about so many things but when their marriage falls apart they cant get support<br><br>The indicators are a blue sky for quality care and outcomes. They will however have the opposite effect of they place further pressure and an exhausted and demoralised workforce and take time away from the important role of creating a safe space to guide patients through the health system, co-ordinate their care, protect and advocate for them in health system that does not always have their best interests at heart.<br><br>practices might get fixated on these indicators and miss other opportunities.<br><br>Not focus only on paper work and neglect patient in the process<br><br>Time time time!!! It will become the inevitable focus at the cost of other care. It will be used as a stick!<br><br>It will be used to devalue and demoralise and to fund general practice and the focus will be shifted to the measurable with an actual loss of care as we all abandon the intangible aspects of care to maintain funding to keep our practices going.<br>Relying on the tool and not engaging with patients<br><br>na focus on remuneration instead of patient care outcomes<br><br>The indicators and measures are very comprehensive, however they are only as good as those GPs who implement them on a consistent basis.<br><br>Unless their is a genuine conversation about the viability of medicare/universal health care and its importance - such tools are not equitable measures and none of the metrics can be used with right utility to bring about meaningful change - and especially if it means more paperwork for the users

		Areas that are not feasible on a practical level would be neglected despite there being value in their contribution to improving quality care.<br><br>Not all practices would utilise the tool<br><br>acceptance of feedback by providers<br><br>Compliance and adherence to using the tool, time, lack of staffing<br><br>It is so hard not to focus on the 2% negative rather than the 98% positive as a GP<br><br>The tool would have to compliment the gaps of what is already being done by the PHN otherwise it will just be another tool of many that may be used to inform quality<br><br>Risks of using this tool could include possible data breaches if online or the possibility of not putting in all the required information.
**Barriers**	Benefits of tool unclear	The benefits of QUEST PHC tool have not been made clear to me how it would benefit general practice.
	Concerns about how data will be used	With sensitivities about such information (demographics) these days - rightfully so - as in the past and is currently still used to exact institutional biases around models of care that still does not support health care and delivery for our diverse population. So I still have reservations about who is collecting demographic and SES data and how that data is being wielded to substantiate funding models and galvanizing blame games.<br><br>Could be used to punish practices seen to be ''underperforming'' but service demographic with more challenging health needs. If publicised, could deter GPs from joining practices where they may be needed most / could cause patients to lose faith in their local practice

		Misinterpretation of data, non contextual use
	Concerns with regard to PRMs	We talk about person centred in our primary care system but seeking feedback from our patients and forming that ideal partnership is still a long way away. I think this is due to making PRM feasibly even though it very relevant many clinicians do not work or see this.<br><br>It (PRMs) would be useful as a screening tool, but there are a few things to consider: - Support the patient's right to refuse to participate - Consider the setting in which the data is collected, to ensure comfort and privacy - There are time constraints to factor in - Only doctors or clinical staff should interpret results<br><br>Patients should be registered to practices . prms reported quaterly by patients - independently and not by practice or during practice visit. And if certain metrics exceed area average - PHN should offer the training and the resources to the practice to remediate.<br><br>**Confusion around PAM meaning**<br><br>Don't know what a Patient Activation Measure score is<br><br>I have never heard of PAM before and so had to look it up. Having no real experience don't feel I can answer the question-so please consider disregarding my answer to question 4.<br><br>Unsure what "patient activation" means and not sure whether applicable. Person centred care and patient team relationship are very important but don't necessarily need to be measured.<br><br>I am not too sure exactly what was meant by Patient Activation Measure scores?? I think next survey maybe clearer language, you are an expert in research and will understand these terms but most clinicians will be a be less clear/certain of what is actually being asked. ... Just my thoughts

		Please don't assume all respondents understand "Patient Activation Measure® scores". It would be nice if you provided a little drop down menu to explain these type of terminology. Thank you.
	Risks of alienating/demoralising practices	as included in previous question - that it risks alienating providers who provide good clinical care in a poor practice environment<br>might not suit all practices - maybe suited to transformational (PCMH) practices
**Perceptions of suitability of the tool for different populations**	Equity of access across different populations	may not capture all relevant measures dependant on location and patient demographics<br><br>Failure to capture the inherent difficulty of different patient populations. Focusing on absolute outcomes rather than relative improvement.<br><br>**Adolescents**<br><br>Adolescents may be accompanied by parent / carer in GP appointment and may result in consumers not declaring accurate history of lifestyle risks eg will an 16 year old declare smoking, substance or alcohol use in front of their parent? It is of questionable value if parent accompanying and leads to misinformed clinical record - perhaps nothing recorded is preferable in this instance. It is of complete relevence for accurate social history and health preventative activities by way of cancer screening and immunisation - we need to strive more to build health literacy in communities so populations are able to make informed decisions about their health. It is not realistic to expect a GP to provide a comprehensive education session with consumer in short time frame - this could be done at a community level to build health awareness, literacy and expectation of improved health outcomes<br><br>It is very challenging to have accurate mental health diagnosis for say a teenager in a crisis - the situation may cause a high level of stress and anxiety related to the situation.

		**Elderly**<br><br>My Health Record requires a level of comprehension and access to technology. Eg it is virtually impossible for an individual with Alzheimer's to have a My Gov Account and MHR - even with Carer with Power of Attorney and Enduring Guardian - for health related decisions.<br><br>**Vulnerable populations**<br><br>Implementation of new processes in general practices is extremely difficult and requires dedicated funding. Involving vulnerable groups in care can be challenging and needs very careful thought.<br><br>Measure p44a - not all patients have a MHR - including refugees or other pt cohorts that may be larger cohorts from some practices.<br><br>**Lack of health literacy**<br><br>We don't have a questionnaire for patients to complete re care = can find one in English, but most of the patients would not be able to read & complete it.<br><br>You must also consider the language skills and health/general literacy of patients when designing these surveys etc.<br><br>**People of non-English speaking background**<br><br>may not capture all relevant measures dependant on location and patient demographics

	Suitability across different geographical areas	My view is that it would be ideal to survey patient experience of health experiences and perception of care, however this would be very challenging to undertake across Western NSW region to encompass remote areas and populations with social disadvantage - many without access to technology and various levels of health literacy and expectations of care. The term patient is no longer considered appropriate language and has evolved to consumers although this is harder to phase out in larger organisations and some clinicians continue to use the word patient.<br><br>It is difficult in remote & regional areas to have access to mental health interventions even if customer has suicidal ideation. there is a lack of support for individuals with substance abuse - there is a lack of access to rehab facilities or interventions such as methodone clinics.<br><br>Remote NSW experiences challenges with access to multidisciplinary team care services. Reasons include whether the service providers are available at a local level or whether people need to travel extensive distance to access allied/medical services in a regional town/city.<br><br>Needs in areas will differ nationally and even locally<br><br>Data could be vary from one region to another Delay in getting response from different regions<br><br>presenting this data without taking into account the challenges or individual districts<br><br>Not understanding regional contexts or general practice. That outcomes are not purely related to the practice but influenced by social, demographc and broader health sector performance<br><br>No understanding of what is happening at Clinic level, broad demographics and diversity from state to state

		Create equity among practices while appreciating the different client needs and complexities.<br><br>as included in previous question - that it risks alienating providers who provide good clinical care in a poor practice environment<br>might not suit all practices - maybe suited to transformational (PCMH) practices<br><br>Differences between each practice and PHN would have to be considered and how each client group differs in their care needs and ability to access care.<br><br>Establishment of standards or benchmarks Take into account regional differences or weighting for particular locations Incentivising practices to take it up in a way that was not seen as punitive Monitoring and reporting Getting the major medical bodies onboard<br><br><br>Mental health services (lack of access to psychiatrist in rural areas)<br><br>The indicators do reflect markers of high quality care but are largely aspirational and contingent on where you work, the billing structure and the quality of education, resources and funding for the upskilling of your work force to deliver on these indicators and their measures. Otherwise I see these indicators which are not contextualised as a means of highlighting difference in a punitive way that seeks to shift blame to individuals rather than the structural constructs that are inherently bias by design.<br><br>Ideally yes however need to be careful as the tool would be reflecting the overall general practice, some practices may score low but have GPs that provide a high level of individual care. Its a hard shift for some to see that overall service to patients includes both the clinical care and the access, records, data analysis and other systems and processes in place in the practice
**Implementation**	Promotion and communication	Quality resources and communication to promote to practices within the region

	Must have buy-in	Qualitative measures are not looked at enough and the role of engagement and trust and transparency would help practices have that conversation about what quality care means. What it requires to deliver care and hopefully frame the conversation in a shared language so everyone is speaking to the same things.
	Carrot not stick	Compliment practices when they are making achievements for patients and practice outcomes. Whatever is to be introduced can't be an extra burden on their already heavy load. It has to be motivational and achievable in small increments for practices to achieve the goals they seek to meet.<br><br>if the tool presented measurements of indicators in a way that would guide practices on what to focus on. if it came with activities/suggestions on how to address. if it is super quick & easy to use<br><br>The tool would be great but beware that it does not become the stick that beats the last ounce of real care out of general practice.
	Staff to be involved	**Staff/ to be involved**<br><br>Availability of a practice nurse COVID safety Staff trained in quality improvement and change management.<br><br>Not a lot of people know about them so more education and understating of the indicators is required in general People also have to report a lot more than they do in order for them to be effective<br><br>Skills in staff in implementing quality improvement. Commitment to the practice to do it long term Reporting and monitoring of results Change management in the practice Attitude and level of engagement of practice manager<br><br>Training staff (clinical and non-clinical) and assigning different roles for them, and ascertaining which indicators to prioritise for our specific patient population.

		Involving the GPs where they need to interact with the process, review the outcome and implement continuous improvement.<br><br>lack of leadership to implement and evaluate. lack of field workers with relevant skill-set.<br><br>Dedicated topic staff available<br><br>Manpower - as need someone dedicated to look after and manage data<br><br>Communication campaign GP champions Support from major medical bodies Apply sound change management principles<br><br>The nurses will most likely use them the most and second to that the general manager would help provide areas for GP's to target specific patients<br><br>As data becomes king - most of these endeavours become feasible but a team needs a team member dedicated to that role of consistent data extraction and analysis , with a whole of team approach supporting the coding and maintaining inputs.<br><br>I think there are still practices who could be involving their team especially nurses more, to provide their patients with patient-centered care and better utilize their time,<br><br>The practice manager would likely be nominated as the staff member responsible for identifying indicators to work on and implementing change.<br><br>i think everyone at the practice would have a responsibility for use of the tool - i think if one person (e.g practice nurse, principle GP or practice manager) would have sole responsibility for using a tool then it would never be used.

		**Training**<br><br>Need to ensure staff buy in and appropriate training to address outcomes from tool with the practice<br><br>Implementing training across sites<br><br>Training in usung tool and education around indicators and their definitions<br><br>Continuous training and support for all staff<br><br>train, guide and assist the practice management to do that.<br><br>Explanation of educational / practice development benefits. Additional staff hours and funding.<br><br>To assist with the implementation of this tool, adequate training and information should be distributed to all Practice Managers and General Practitioners about the changes.<br><br>clear, concise readily available training<br><br>Training and support services (manuals, FAQs)<br><br>Walk-throughs, webinars, on-site training.<br><br>Education; resources; guides<br>Training staff and practice team, Quarantined time, Financial<br><br>Awareness, training, education and the benefits as to why it would be of benefit.<br><br>Clear, concise and readily available training

		Introduction and training in the benefits and use of the tool. Webinars would be good as is flexible and time efficient.<br><br>PHN to provide training to general practice staff<br><br>Education around the tool for PHN Practice Support and digital team. Training the PHN Practice Support Team. Advocating to General Practice<br><br>Again as my answer above adequate training and information should be distributed to all who will assist with the rollout of this tool.<br><br>Training and support to assist general practice staff<br><br>education on use and implementation of the tool/s to ensure standardised results<br><br>PHN implementation staff would need to be well equipped in their knowledge of how the tool is going to benefit the practice and their patient population. PHN staff would need to be confident the application is going to be of benefit for improved health outcomes otherwise there is a risk to the PHN<br><br>Better understanding of general practice. Extensive education / explanation of and consultation with stakeholders. Gradual implementation rather than the usual precipitous deployment of new policy.<br><br>Ensuring that training and information being distributed to everyone.<br><br>Education and greater understanding of demographics at ground roots level<br><br>More focus training - i would say complusory for GPs - they get lasy<br><br>Coaching or education regarding optimizing practice workforce, I think there are still practices who could be involving their team especially nurses more, to provide their patients with patient-centered care and better utilize their time,

		Explanation of educational / practice development benefits. Additional staff hours and funding.<br><br>Better trained staff on different practice software.<br><br>Quick guides with ideas on how to achieve indicators<br><br>I would assume possibly disinterest from certain practices, having to educate as well as implement around the fundamentals of Clinical and Corporate Governance.<br><br>Training staff (clinical and non-clinical) and assigning different roles for them, and ascertaining which indicators to prioritise for our specific patient population.<br><br>Not a lot of people know about them so more education and understating of the indicators is required in general People also have to report a lot more than they do in order for them to be effective
	Integrate into current normal workflow or improve	currently these measure are easily collected and should not change<br><br>A tool that is user friendly and that could be incorporated into everyday work process than it could be fundamentally used by most practice staff<br><br>A tool like this could be utilised to streamline care within General Practices.<br><br>Implement via various avenues by several parties: PHNs, GP practices/ practice owners and practice managers<br><br>If burdensome or inflexible to respond to particular patient profiles and needs will not be used.<br><br>I think clinical audit tools can be quite saturated in the market and may not get a large uptake. mThere are also privacy concerns if the tool is collecting practices data and if it

		is easy to integrate general practice day to day.integration with data analysis tools (pencat, polar, primary sense) would assist in benchmarking.<br><br>As day to day part of all management. To inform usual care - for assessment and monitoriing<br><br>breaking the indicators down into categories and then further categorising in terms of what can be easily achievable. Integration with data analysis tools (pencat, polar, primary sense)<br><br>In my view the tool would need to run in the background to collect data without the need for additional workforce input. If the QUEST PHC was developed as an extraction tool with fields that were inline with current medical software fields then i could see this tool implemented successfully.<br><br>Ideally the tool would be beneficial if it is integrated with their clinical and billing systems. Again user friendly, incorporate into everyday work process. If this is not the case than it would come down to time and who would be expected to access it. If too much for a practice the tool may not be used. Would practice staff require training and support in using the tool.<br><br>Actually using it as part of routine and workflows<br><br>The different systems used and the limitations about how this integrate between providers and within the overall health system while keeping the patient in mind at the centre of that care.<br><br>Information sharing and lack of technological framework to keep pace with consumer needs.<br><br>The tool can be used in the same way as CAT4 or POLARGP, where data is extracted from the clinical software and measured against the set of indicators.

		The tool be interoperable within general practice software if possible. User friendly and for processes to be developed as everyday scope of work rather than additional work. Training and support<br><br>software included prompts and extraction that save time, deliver more intuitive engagement for clinician and patient while generating meaningful value for sustainability of primary care and universal health care and access. Change agents that are provided free from external funding to recommend change and provide the support to bring in that change rather than then given another report that a busy practice needs to find the time to make along with meeting accreditation and MBS requirements.<br><br>Having a tool that may be compatible with practice software or easy to access/work through<br><br>making it as streamlined as possible and even integrating into the current patient medical system to make it seamless<br><br>must be integrated in the workflow electronically. Not to add to the burden.<br><br>I think soft ware that automates the recipes, processes and prompts- where there are prompts obstructive to certain processes without the necessary inputs can condition practices and behaviours to inform the feasibility of outcomes of data collection. I do however believe that covid will be become obstructive to such investigations such as spirometry and where patient contact is paramount. I do believe though that patients are more ready for wearable technologies and engagement with these wearable will give healthcare teams greater access to almost "free" data<br><br><br>**Must be Easy to use**<br><br>It's a good concept, but it needs to be simple enough to encourage stakeholders to involve

		Develop specific software not more paperwork<br><br>Any measure has to be doable and be seen as sensible by general practice - we don't want a wish list - start small and grow.<br><br>As mentioned previously, this tool any tool that will be used in general practice has to be user friendly, interoperable with clinical software if possible and not a burden for time management for the practice staff utilising the tool. If the tool can support all measures mentioned for patient outcome but also to support general practice as a small business.<br><br>The tool needs to be easy to use, maybe involve prompt.<br><br>Again if tool is user friendly (as above) than it could very well support quality of care and especially QI activities that are now very much a process in general practice. The tool could streamline processes in general practice<br><br>not much. Just need to make it simple so everyone can understand the use<br>if the tool presented measurements of indicators in a way that would guide practices on what to focus on. if it came with activities/suggestions on how to address. if it is super quick & easy to use<br><br>***Other IT tools that could potentially compliment/feed data into QUEST PHC***<br><br>Dr's use safe script in the practice to monitor and check scripts patient has had recently<br><br>Measure O78a - this is possible through our data linkage project called Lumos - we are already providing reports on this to our practices.

		This is new space we have LUMOS and now we are seeing more richness in this data but needs to be ongoing and reports simplifed to provide the information back to GP and also used for quality improvement measures<br><br>While most of the indicators are relevant, some of them are dependent on external systems which vary enormously. E.g., hospital use of notifications, MHR, communication generally...there would need to be significant investment in tertiary system use to align and connect with primary care.
	Implement an appropriate number of indicators	It is also time /resource consuming to report on a large number of measures.<br><br>Any measure has to be doable and be seen as sensible by general practice - we don't want a wish list - start small and grow.<br><br>Very comprehensive suite. Need to cone down the measures.
	Need a benchmarking/ standardisation component	Standardising measures and ensuring they can be applied to all general practices (from solo to large practices and also for metro to rural or very remote). Some practices do find RACGP 5th Edition standards favour larger practices and making it universal for any practice no matter size or location.
	Need accurate datasets	**Data quality/accuracy from clinicians**<br>Poor or incorrect understanding of practices capacity to measure indicators resulting in flawed data<br><br>Risks of using this tool could include possible data breaches if online or the possibility of not putting in all the required information.<br><br>There can be some risks such as inaccuracies when using the tool<br><br>Can be difficult to implement in some areas and relies on the user coding things correctly

		Obtaining meaningful data from GPs<br><br>Unsure. Just don't know how you measure the intangible accurately!!<br><br>Many opinions and variables and not being able to satisfy everyone<br><br>**Data cleaning required**<br>i would also like to highlight the importance of data collection/coding and cleaning for general practice - there are limitations within the way practices use software to their full capacity that can often impact recorded measures. there is still a large gap in terms of data cleaning & coding processes within general practice.<br><br>…this is a HUGE ONE data cleaning to reflect true measures the data extract recipes from 3rd party data extraction tools difficulties of the tool taking into consideration general practice a small business<br><br>**Concerns about representiveness of data collected by the tool**<br>Because not all practices will use the tool, the results would be an inaccurate indicator across the PHN<br><br>May not get an accurate representation of data sets<br><br>If not indicators not measured and recorded consistently by all practices and supported by PHN's then data may be flawed and results inaccurate<br><br>Incomplete data or skewed data based on who is responding
	Data linkages needed	Need to link it with morbidity and mortality data and use of health services

	Need to overhaul the healthcare system	Many more - requires financing for training in QI, workflows then financing for support to implement improvements, then support for better integration of care in primary/secondary and tertiary sectors, then shared responsibility across the whole health sector for health improvement
	Need to be supported by PHNs to implement	engagement and support provided to clearly help practices to move up in their scoring on such a tool<br><br>Challenges at a PHN level could include not having the full history of the patients at their ready so they are able to run the appropriate reports.<br><br>We are already doing a lot of this work - but practices would need support<br><br>Prepare to provide assistance and support<br><br>None, just need to be able to assist practices in implementing them<br><br>Managing the transition of practices using other tools to this one.<br><br>Supporting implementation of the tool in general practices that are already extremely busy and short staffed would be challenging at this time. PHNs strive to support QI as a component of BAU and are well-placed to offer support at a local level for practices that implement QUEST PHC.<br><br>PHNs would need to work with General Practice on this<br><br>INVOLVE ALL PHN<br><br>PHN staff with relationships within practice to be involved<br><br>PHNs are well placed to implement tools across Australia

	Suggestions of features for the tool	A dashboard highlighting the measures would be useful.<br><br>Need a centralised data based / possibility cloude system
	Evaluation of the tool	Reports and data updates of how the tool has been working
	Make it a national tool	Make performance a national performance indicator
	Who will be the users	Encouraging the clinical team (The nurses as well as the doctors) to use it, encouraging it to be used frequently
