# Supplementary material for: Indicators of high-quality general practice to achieve Quality Equity and Systems Transformation in Primary Health Care (QUEST-PHC) in Australia: a Delphi consensus study
Source: PLoS One. 2025 Sep 5;20(9):e0327508. doi: 10.1371/journal.pone.0327508 (PMC12412998; doi:10.1371/journal.pone.0327508)
Supplement: Attachment — Submitted filename: rebuttal letter 20250530.docx [file pone.0327508.s006.docx]

Dear Editor,

On behalf of my co-authors, I submit below our response to the reviewers. We thank both reviewers for their time. We greatly appreciate it.

Regards,

Phyllis Lau, 29 May 2025

| **Journal and reviewers’ comments** | **Authors’ responses** |
| --- | --- |
| Reviewer #1: |  |
| The study provides invaluable insights into the critical factors influencing primary healthcare in Australia, particularly in achieving quality, equity, and systems transformation. The comprehensive approach, involving the Delphi consensus method, contributes significantly to the field and serves as an essential resource for those working in primary care and health policy. | We thank the reviewer for their generous comments. |
| General Comments: • The study mentions achieving "wider consensus," but clarifying who the "wider" audience refers to and whether the findings could be generalized to settings beyond Australian general practice would be beneficial. | This information is already in the manuscript. The wider consensus was achieved “with general practice and PHN staff” (line 130) “in four states” (line 159) on the indicators and measures developed “based on the literature and extensive consultation with PHNs in western Sydney” (line 115).  We nevertheless agree that these needed to be clearer. The last sentence in Introduction is now amended to “This paper reports on the results of the Delphi consensus study conducted, as part of the content validation process, to establish wider consensus with general practice and PHN staff across four states on the relevance and feasibility of the identified suite of indicators and measures for the ~~wider~~ Australian context.” (line 128-132)  We have also added a sentence to the end of Conclusion – “Generalisability of the QUEST PHC tool beyond Australian general practice should also be investigated.” (line 655-656) |
| Methods Section and Setting: • I would suggest adding the protocol of this Delphi consensus study (briefly), including the justification of the methodology.  • It’s beneficial for the readers to add the characteristics of the 162 PHNs, their geographical locations, and the populations in their regions (briefly).  • Please justify the sample size and if any calculations were used to determine it.  • The description of how consensus was determined (≥70% agreement for relevance and feasibility) is good, but providing more context on the threshold chosen for consensus would add clarity. Why was this agreement threshold selected, and is this typical in Delphi studies in healthcare? | - A summary of the protocol (reference provided), including justifications for any variations, is already written in considerable details in the Methods section. - Only eight, not 162, PHNs were involved, and a brief description of their characteristics including populations are already in Methods under Settings. We have now added their geographical locations by naming the four states (where the eight PHNs are located) in the same section – “The Delphi study was undertaken in four states (New South Wales, Queensland, Victoria and Western Australia) in Australia across regions of the eight PHNs” (line 159-160). - We have now amended the Sample Size section in Methods clearer – “Formal sample size calculations are not required in the Delphi methodology. The study therefore aimed to recruit a minimum of 80 participants. The median number of participants reported for content validation in previous Delphi studies involving the selection of healthcare quality indicators was 17.^31^ Following consultation with the Project Governance Group and considering the participation of a general practice and primary health care expert group rating on 128 measures, particularly during the COVID-19 pandemic, the study therefore adopted a pragmatic approach and aimed to recruit a minimum of 80 participants for Round 1, with an anticipated retention rate of 40-45% in subsequent rounds to attempt to meet the minimum sample size requirement.” (line 166-174) - The reason for selecting the 70% threshold is already provided in line 216-217 and 219-221. We have now added a sentence to explain that there is no standard or typical threshold in the Delphi methodology – “The levels of consensus in the Delphi methodology vary depending on size of the expert panel and the aim of the research.” (line 215-216) |
| Data Collection: • It would be beneficial if you could clarify if the consent form has been submitted and how and where it was obtained. | This information is already provided in line 139 under Ethics Approval, and in line 178-180 under Participants and Recruitment. |
| The results: • The results mention that 19 measures were "slightly less feasible" than others. It would be helpful to elaborate on what factors contributed to these measures being rated as less feasible. More specific examples would make the results more actionable for readers. | The possible factors contributing to the 19 measures being rated as less feasible are already discussed in line 564-567 in Discussion. |
| Conclusions: • The conclusions are firm but could benefit from further exploration of the practical implications of the results. Specifically, how might the findings influence policy or the future implementation of the QUEST-PHC indicators in general practice? Additionally, while the feasibility of some measures needs further consideration, providing examples or suggestions on how to address these feasibility concerns would strengthen the conclusion. | Thank you to reviewer for their suggestions to strengthen the conclusions. Both points are discussed within Discussion, but we agree it could be highlighted again in Conclusions.  We have now added to line 642-650: “This set of indicators and measures has the potential to define high-quality general practice, and inform primary health care quality improvement and funding reforms, aligning with Australia’s Primary Health Care 10-Year Plan 2022-2032 to achieve person-centred primary health care and providing the evidence for a VPR platform to incentivise quality person-centred primary health care. However, further consideration about feasibility in the context of systemic, GP-specific and patient-specific barriers, including issues with current health system constraints, existing health data linkage, and general practice buy-in, capacity and time, is required for some of the measures.” |
| Reviewer #3: |  |
| the title the authors and and a statement in the abstract section quite different from that of the concept stated in the title, in my opinion either need a modification. the concurrent mixed approach is purely a quantitative and qualitative method, but you authors did not claim about the quantitative one; this is purely a qualitative so why concurrent mixed methods is applied? also it lacks about the concepts Anonymity, Iteration, Controlled Feedback, and Statistical Stability of Consensus; except a few stated about iteration; even steps in the Delphi process are not well defined. Also there is nothing about indicator development (If I missed ..). it lacks the evidence to set a minimum of the numbers of participants. | We thank the reviewer for their comments but would nevertheless refute them.  The title stated a Delphi study which is what we described in the manuscript. The Delphi study used a mixed-methods approach which we have also exhaustively described in the manuscript. We do not quite understand the reviewer’s comment that “this is purely qualitative”. In this mixed-methods study, we integrated both quantitative and qualitative results to explain, supplement or raise questions about what we found. Please see Methods, Results and Discussion sections to see how this mixed-methods Delphi study is described.  The elements of the Delphi process are clearly described in the manuscript. Study participants were anonymised (line 196-197)(Anonymity) in three rounds of survey where participants were presented with items to rate and comment in rounds 1 and 2, and then re-rated items that did not reach consensus and provided feedback to open-ended questions in round 3 (line 222-248)(Iteration and Controlled Feedback). The quantitative analysis is described in Methods (line 249-272), and extensively in Results (line 301-417)(Statistical Stability of Consensus).  The development of the indicators is described in Methods (line 112-124) and references were cited for further information.  Sample size justification is provided in Methods (line 165-174) now with modifications following Reviewer 1’s comment. |
| Pg 10: this is quite different from that of the concept stated in the title, in my opinion either need a modification | The reviewer’s comment here refers to the objectives stated in the abstract “This study aimed to achieve wider consensus on the relevance and feasibility of the Quality Equity and Systems Transformation in Primary Health Care (QUEST-PHC) indicators and measures developed for Australian general practice”. The Delphi methodology is then clearly stated in the Abstract’s Methods. We respectfully disagree that the concept of the Abstract’s Objective is different to that stated in the title “Indicators of high-quality general practice to achieve Quality Equity and Systems Transformation in Primary Health Care (QUEST-PHC) in Australia: a Delphi consensus study”. |
| Pg 12: this is unnecessary punctuation and it is best if it is cited line 57-60 | We could not quite find the “unnecessary punctuation” that the reviewer referred to. We in fact feel we have missed an open quotation mark, now added to line 58.  The two references are already cited in line 65. |
| Pg 15: this approach is purely a quantitative and qualitative method, but you authors did not claim about the quantitative one; this is purely a qualitative so why concurrent mixed methods is applied?  also it lacks about the concepts Anonymity, Iteration, Controlled Feedback, and Statistical Stability of Consensus; except a few stated about iteration.  even steps in the Delphi process are not well defined. Also there is nothing about indicator development (If I missed ..) | This comment is the same as the reviewer’s first comment. Please refer to our previous response. |
| Pg 16: hopefully needs a reference | Reference 25 for the development of the indicators and measures including the literature review conducted is already cited in the next line (line 145). |
| Pg 16: what is the relevance in the article section, if it is a must it is best to state in the setting section. | We respectfully disagree with the reviewer.  The Project Governance section outlining the key stakeholders involved in the implementation, dissemination and collaboration of a research aiming to reach consensus on quality indicators in Australian general practice is very relevant. The environment surrounding this research was sensitive, often even political, and this section makes it clear that we have broad buy-in from all relevant stakeholders, which was critical for the success of this project. |
| Pg 17: the evidence to set a minimum of the numbers of participants? | Again, this comment is the same as the last part of the reviewer’s first comment. Please refer to our previous response. |
| Pg 17: it is good to state about how this indicators were developed; if I am not missed | Again, this comment is the same as that in the reviewer’s first comment. Please refer to our previous response. |
| Pg 21: A response rate for each round??????? overall is about 64%.  To maintain rigour, as a guide it is suggested that the response rate of each Delphi round should not fall below 70% (Kilroy and Driscoll 2006). No subsequent rounds should be launched if the response rate  of the previous round is low. | With respect, the reference cited is almost 20 years old.  A much more pragmatic orientation is preferred in the Delphi Methodology nowadays.^1,2^ Although Brady (2015) did not specifically provide recommendations on retention rates, he alludes to the creation of “formal practice theory grounded in the practice experience of community organizing practitioners working in community settings”.^1^ Shang (2023) acknowledges the issues of low retention rates and provides pragmatic approaches.^2^  In consultation with our Governance Group, we knew that 70% retention in a general practice and primary health care expert group rating on 128 measures, particularly during the COVID-19 pandemic, would be unachievable. Instead, we focused on encouraging participation through reminders and personalised communication, extending deadlines, and acknowledging participant contributions.  The real-world challenges we encountered have been outlined multiple times in line 169-174 (in our amendments to address Reviewer 1’s comments), line 199-200 and line 631-634.  *^1^Brady, S. R. (2015). Utilizing and Adapting the Delphi Method for Use in Qualitative Research. International Journal of Qualitative Methods, 14(5).* [*https://doi.org/10.1177/1609406915621381*](https://doi.org/10.1177/1609406915621381) *(Original work published 2015)*  *^2^Shang, Zhida (2023). Use of Delphi in health sciences research: A narrative review. Medicine 102(7):p e32829, February 17, 2023. DOI: 10.1097/MD.0000000000032829* |
| Pg 24: it is best to say something about the dropouts if the fate of one/third of the participants is known | As explained in our previous response, the reasons for attrition are already outlined in line 169-174, line 199-200 and line 631-634. |
| Pg 24: if it is reported degree of variation should be stated i.e.., SD | Participants’ demographics were analysed descriptively (Methods line 250). We did not calculate SD. |
| Pg 26: for ease understanding it is best to describe the average number of raters for each attribute for the overall three rounds unless it is very difficult to comprehend this table. Hopefully all measures of central tendency is nor as such important rather it made confusion; as researchers decision should be made to use either; it makes complicated | The number of raters for each attribute is already clearly stated in the second row in Table 4.  The reviewer referred to ‘normalisation’ in their comment. The primary purpose of the Delphi methodology is to generate consensus in a group of experts on a particular topic or issue. It does not necessarily strive for ‘normalisation’. In this study, we did not transform our data into a normal distribution, instead we have chosen to measure agreement using percentages of agreement or changes in ratings between the different levels of agreement. We hope this answer satisfies the reviewer. |
| Pg 28: this is not scientifically sound, mean with SD; or Median with range should be reported with a possibility of min. Vs Max. score report. | We have stated in Methods we have chosen to use ranges and skewness to demonstrate the distribution of our data. We have now added to this statement – “The analysis in the original protocol also included interquartile ranges and associated group rankings, but due to the skewness of the scores, these statistics and standard deviations or median ranges did not provide added value, so instead ranges and skewness were calculated to demonstrate the distribution of the data.” (line 260-263) |
| Pg 33: the same comment as of table 4 | Our response is similar to that provided for Table 4 – The number of raters for each attribute is already clearly stated in the second row in Table 6. |
| Reviewer #4: |  |
| Thank you for this well written paper that addresses an important area with the potential to advance healthcare equity in primary care. Here are a few of my observations: | We thank the reviewer for their generous comment. |
| The abstract mentioned using thematic analysis, while the main paper also mentioned using content analysis. Could you clarify whether it is thematic analysis? or content analysis was used to gather data into themes? It would be helpful to provide more clarity on this.  If content analysis was used and the qualitative part aimed to identify additional indicators or measures, it would be helpful to specify whether any new indicators or measures were identified. While content analysis doesn’t always require reporting counts, it might be useful to consider quantifying the codes/themes to provide a clearer understanding of how frequently a particular item appears in the data. This could be especially relevant if any recommendations have or will influence the refinement of the indicators or measures. Additionally, a brief statement on how the qualitative findings influenced the indicators or measures, beyond the barriers and facilitators discussed under the discussion section would be helpful. | We thank the reviewer for picking this up. We confirm that we only conducted thematic analysis in this study.  We agree with the reviewer about the usefulness of content analysis in understanding the relevance or influence of any recommendations. Our original intention was to also conduct content analysis to attempt to determine the presence of certain concepts and ideas. However, the diverse nature of the qualitative data made this exercise near impossible. We have now deleted the sentence about content analysis in Methods (line 286-288).  We already have a sentence in the ‘strengths’ paragraph in Discussion that articulates how the qualitative findings provided “a comprehensive picture of the validity of the indicators and measures as well as informing their future implementation in the real world” (line 625-628). Please let us know whether this will suffice. |
| While the process is well detailed, it would be helpful to indicate the reporting guidelines used for this study. | We have added a sentence under Supporting Information on the use of the Conducting and REporting of DElphi Studies (CREDES)^3^ guidelines to guide our reporting (line 673-674). Again, please let us know whether this will suffice.  *^3^Jünger S, Payne SA, Brine J, Radbruch L, Brearley SG. Guidance on Conducting and REporting DElphi Studies (CREDES) in palliative care: Recommendations based on a methodological systematic review. Palliat Med. 2017 Sep;31(8):684-706.* |
